# The induction of autophagy against mitochondria-mediated apoptosis in lung cancer cells by a ruthenium (II) imidazole complex

**DOI:** 10.18632/oncotarget.13032

**Published:** 2016-11-02

**Authors:** Lanmei Chen, Guodong Li, Fa Peng, Xinming Jie, Guangzhi Dongye, Kangrong Cai, Ruibing Feng, Baojun Li, Qingwang Zeng, Kaiyi Lun, Jincan Chen, Bilian Xu

**Affiliations:** ^1^ School of Pharmacy, Guangdong Medical University, Zhanjiang, 524023, China; ^2^ Analysis Centre of Guangdong Medical University, Zhanjiang, 524023, China; ^3^ State Key Laboratory of Quality Research in Chinese Medicine, Institute of Chinese Medical Sciences, University of Macau, Macau, 999078, China; ^4^ Guangdong Key Laboratory for Research and Development of Nature Drugs, Guangdong Medical University, Zhanjiang, 524023, China

**Keywords:** ruthenium imidazole complex, cytotoxicity, apoptosis, autophagy, reactive oxygen species

## Abstract

In the present study, it was found that the ruthenium (II) imidazole complex [Ru(Im)_4_(dppz)]^2+^ (Ru1) could induce significant growth inhibition and apoptosis in A549 and NCI-H460 cells. Apart from the induction of apoptosis, it was reported for the first time that Ru1 induced an autophagic response in A549 and NCI-H460 cells as evidenced by the formation of autophagosomes, acidic vesicular organelles (AVOs), and the up-regulation of LC3-II. Furthermore, scavenging of reactive oxygen species (ROS) by antioxidant NAC or Tiron inhibited the release of cytochrome *c*, caspase-3 activity, and eventually rescued cancer cells from Ru1-mediated apoptosis, suggesting that Ru1 inducing apoptosis was partially caspase 3-dependent by triggering ROS-mediated mitochondrial dysfunction in A549 and NCI-H460 cells. Further study indicated that the extracellular signal-regulated kinase (ERK) signaling pathway was involved in Ru1-induced autophagy in A549 and NCI-H460 cells. Moreover, blocking autophagy using pharmacological inhibitors 3-methyladenine (3-MA) and chloroquine (CQ) enhanced Ru1-induced apoptosis, indicating the cytoprotective role of autophagy in Ru1-treated A549 and NCI-H460 cells. Finally, the *in vivo* mice bearing A549 xenografts, Ru1 dosed at 10 or 20 mg/kg significantly inhibited tumor growth.

## INTRODUCTION

**Figure 1:**
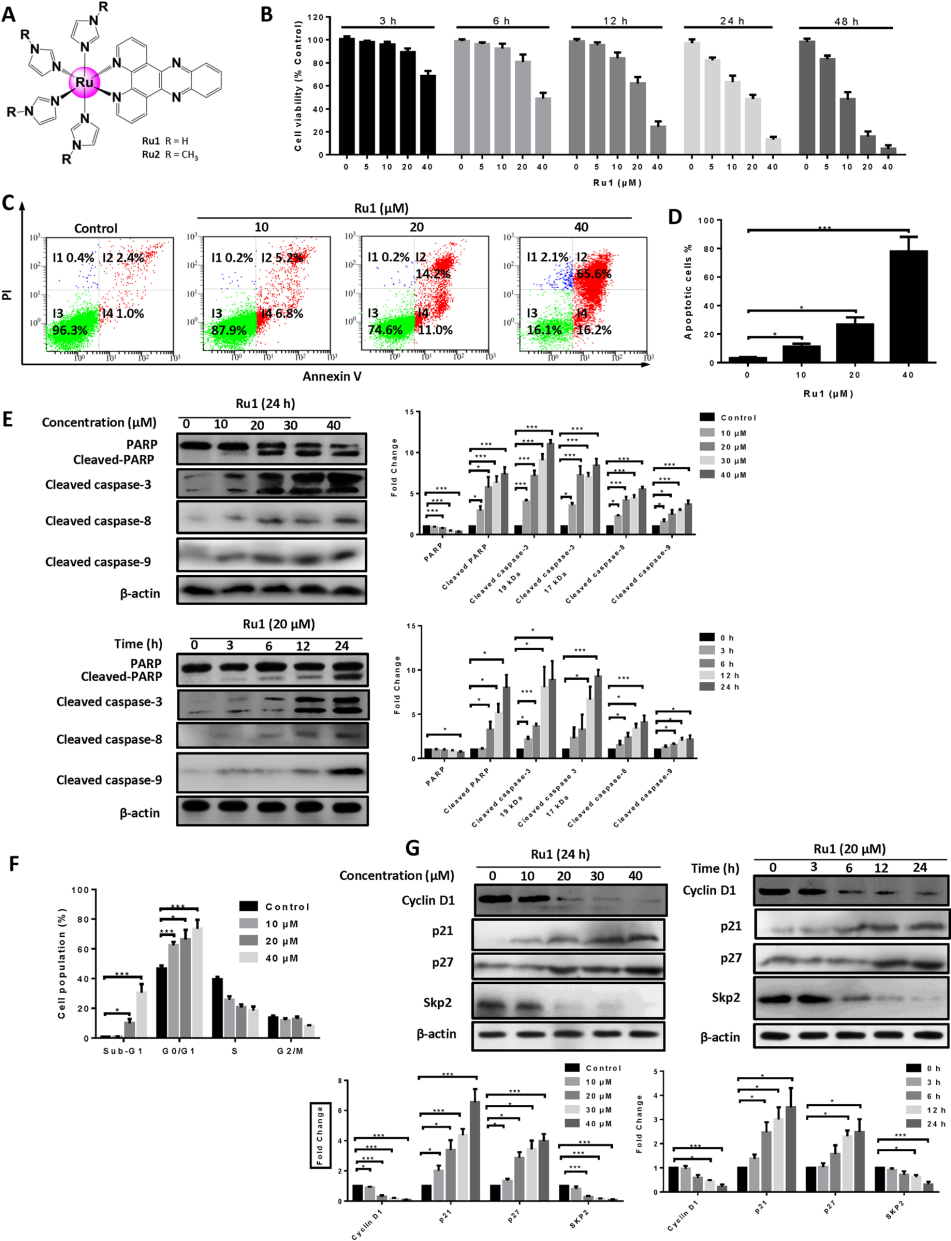
Ru1 induces growth inhibition and apoptosis in A549 cells (**A**) Structures of ruthenium imidazole/methylimidazole complexes Ru1 (R = H) and Ru2 (R = CH_3_). (**B**) The A549 cell viability was measured by MTT assay after Ru1 treatment for 3, 6, 12, 24 and 48 h. (**C** and **D**) A549 cells apoptosis was detected by annexin V/PI assay after co-incubation with 10, 20 and 40 μM of Ru1 for 24 h. (**E**) The expression level of cleaved caspase-3, cleaved-PARP, cleaved caspase-8 and cleaved caspase-9 were manifested in a time- and dose-dependent manner with Ru1 treatment. Right, quantification of the bands normalized by β-actin. (**F**) Cell cycle distribution was performed after co-incubation with 10, 20 and 40 μM of Ru1 for 24 h. (**G**) The expression levels of Cyclin D1, p21, p27 and Skp2 were evaluated in a time- and dose-dependent manner with Ru1 treatment. Below, quantification of the bands normalized by β-actin. Results were represented as mean ± SD (*p < 0.05, ***p < 0.001).

As potential anticancer agents, ruthenium compounds show low toxicity to normal cells and multiple anticancer mechanisms [1]. Two typical Ru(III)-drugs [ImH][trans-RuCl_4_(DMSO)(Im)] (NAMI-A) and [IndH][trans-RuCl_4_(Ind)_2_] (KP1019) have successfully entered clinical trials [2–4]. However, more detailed investigation of the antitumor mechanism of these ruthenium compounds still remains largely speculative [5–9]. In the last few years, our investigations have been focus on the Ru(II)-imidazole/methylimidazole compounds including their design, synthesis, structural modification, biological activity, and underlying behaviour mechanisms [10–15]. In previous studies, Ru(II) methylimidazole complexes, with the general formula [Ru(MeIm)_4_(L)]^2+^ (L = dpq, dppz, iip, tip, cpip), showed strong DNA-binding affinities and some antitumor activities [10–14]. It is worth noting that the homologous complex Ru1 (Figure 1A) bearing imidazole as the ancillary ligand synthesised in our laboratory, differs from other ruthenium (II) methylimidazole complexes in the circular dichroic spectrum of DNA–complex adducts, and so on [10]. It was therefore speculated that Ru1 has a different anti-proliferative effect on tumour cells to other ruthenium methylimidazole complexes.

Autophagy is an evolutionarily conserved stress-response mechanism which often occurs in cancer therapy [16]. However, the role of autophagy in cancer therapy is still unclear. In several scenarios, autophagy can antagonise cancer cell death (suppresses apoptosis) as a cytoprotective mechanism, thus meaning that autophagy inhibition could be used in cancer therapy as an adjuvant therapeutic agent [17–20]. However, in other conditions, autophagy can also lead to cellular demise itself, that is autophagic cell death [21]. Hence, elucidating the functional roles of the influence of autophagy was deemed important for cancer therapy. As for the role of autophagy induced by ruthenium complexes, Tan and co-workers have demonstrated that a series of Ru(II)-β-carboline complexes could simultaneously induce apoptosis and autophagy in tumour cells, and both the apoptosis- and autophagy-inducing activities are associated with ROS accumulation [9]. However, the underlying mechanisms of Ru(II)-induced autophagy have not been evaluated, especially the roles of ROS and mitochondria in Ru(II)-triggered autophagy.

In this work, the underlying mechanism of the antitumous effect of Ru1 in lung carcinoma was explored, and the relationship between apoptosis and autophagy was investigated. For comparative purposes, the Ru(II)-methylimidazole complex [Ru(MeIm)_4_(dppz)]^2+^ (Ru2, Figure 1A) with a similar structure to Ru1 has been also synthesised and characterised [10]. We found that Ru1 induced growth inhibition and apoptosis, which was partially caspase 3-dependent by triggering ROS-mediated mitochondrial dysfunction in A549 and NCI-H460 cells. Moreover, our results demonstrated that Ru1 could induce autophagy in A549 and NCI-H460 cells, and autophagy inhibition could result in the enhancement of caspase 3-dependent apoptosis. Additionally, our results indicated that an ERK signaling pathway was involved in autophagy induced by Ru1 in both A549 and NCI-H460 cells. Altogether, these findings suggested that combination of ruthenium (II) imidazole complex Ru1 and autophagy inhibitors could provide a potential approach in the treatment of lung cancer.

## RESULTS

### Ru1 induces growth inhibition and apoptosis in A549 and NCI-H460 cells

Firstly, the *in vitro* cytotoxicities of Ru1 and Ru2 against five selected human cancer cell lines (lung adenocarcinoma cell A549, human lung cancer NCl-H460, hepatocellular carcinoma HepG2, breast cancer MCF-7 and cervical cancer HeLa) and one normal cell line (human bronchial epithelial cell HBE) were assayed by using 3-(4,5-dimethylthiazol-2-yl)-2,5-diphenyltetrazolium bromide (MTT) assay. Cisplatin has been employed as a positive control. As shown in Table 1, both Ru1 and Ru2 exhibited broad spectrum inhibition of human cancer cells. Notably, Ru1 displayed higher cytotoxicity than Ru2 in five tested cancer cells, which was corresponding to their order of the DNA-binding affinities reported in our previous work [10]. The differences of the electronic and geometry structures between two ruthenium complexes lead to the differences of DNA-binding affinities, which may result in different anti-proliferative activities of Ru1 and Ru2 [10, 15]. In addition, more importantly, compared to cisplatin, Ru1 and Ru2 exhibited much lower toxicity to normal cells. These results indicated that Ru1 and Ru2 had high selectivity between cancer cells and normal cells.

**Table 1:** IC_50_ values (μM) of Ru1 and Ru2 against the selected human cancer cell lines and normal cell lines (HBE)#

Complexes	IC_50_ (μM)					
	A549	NCl-H460	HepG2	MCF-7	HeLa	HBE
**Ru1**	21.24 ± 1.24	23.10 ± 3.2	26.15 ± 1.52^a^	46.17 ± 3.27^b^	40.17 ± 3.27^b^	176.47 ± 13.41^b^
**Ru2**	46.52 ± 3.84^d^	51.23 ±3.81^d^	42.54 ± 3.45^d^	63.41 ± 4.56^c^	62.70 ± 3.74^d^	191.54 ± 10.24
**Cisplatin**	26.71 ± 1.47^c^	29.92 ± 2.97	28.22 ± 2.19	34.14 ± 2.36^b^	20.47 ± 2.08^b^	22.53 ± 2.47^b^

# Drug-treatment period was 24 h. Different cells were treated with tested compounds vs. A549 cells,

a *p* < 0.05,

b *p* < 0.001; homologous cells were treated with various complexes vs. Ru1-treated cells,

c *p* < 0.05,

d *p* < 0.001.

Each data represents the mean ± SD of at least three independent experiments.

Since the A549 cell was especially sensitive to Ru1, with a lower IC_50_ than that of Ru2, it was thus chosen as a cell model to further explore the mechanism of anti-tumor. In addition, as shown in Figure 1B, Ru1 decreased cell viability in a concentration- and time-dependent manner. Annexin V-FITC/PI staining was performed to further confirm the nature of cell death induced by Ru1, and the result was analysed by using flow cytometry. Figure 1C and 1D showed that pre-incubation of A549 cells with different concentrations of Ru1 for 24 h enhanced the percentage of apoptotic cells. Besides, the results of western blot assay in Figure 1E illustrated that the expression levels of cleaved-PARP, cleaved caspase-3, cleaved caspase-8 and cleaved caspase-9 increased in a dose- and time-dependent manner, which suggesting Ru1-induced apoptosis in A549 cells, and both extrinsic and intrinsic apoptosis pathways were involved.

Secondly, the effect of Ru1 in A549 cell cycle distribution was performed by flow cytometry analysis after being stained with PI. Figure 1F showed that the cells in the sub-G1 phase in these Ru1-treated groups significantly increased when compared with DMSO-treated controls, indicating that Ru1 could induce cell death in A549 cells. In addition, upon Ru1 treatment, the number of cells in the G0/G1 phase increased, with reduced cell counts in the S phase, in a dose-dependent manner when compared with DMSO-treated controls, indicating that Ru1 could induce G0/G1 arrest. Furthermore, western blot analysis in Figure 1G illustrated that A549 cells treatment with Ru1 down-regulated the level of Cyclin D1 and Skp2, but up-regulated the level of p21 and p27 in a time- and dose-dependent manner. Cyclin D1 is a cell cycle regulator essential for the G0/G1 phase, and expression of Cyclin D1 correlates closely with development and prognosis of cancers [22]. p21 and p27 are two well-known CDK inhibitors, up-regulation the expression levels of those can block G1-S transition.

To avoid single cell line bias, one more cell line, *i.e.* NCI-H460, has been employed to investigate the antitumor effect and apoptosis of Ru1, and the corresponding results are presented in Supplementary Figure S1. All of these findings demonstrated that Ru1 could induce growth inhibition and apoptosis in A549 and NCI-H460 cells.

### Ru1 induces mitochondrial dysfunction

Mitochondrion, which plays an important role in apoptosis, can release pro-apoptotic factors such as cytochrome *c*, apoptosis-inducing factor (AIF), and endonuclease G [23]. Mitochondrial dysfunction is determined by measuring changes in mitochondrial membrane potential (MMP). The MMP in Ru1-treated A549 and NCI-H460 cells were investigated with 5,5′,6,6′-tetrachloro-1,1′,3,3′-tetraethylbenzimidalylcarbocyanine iodide (JC-1) as fluorescent probe [24].

**Figure 2:**
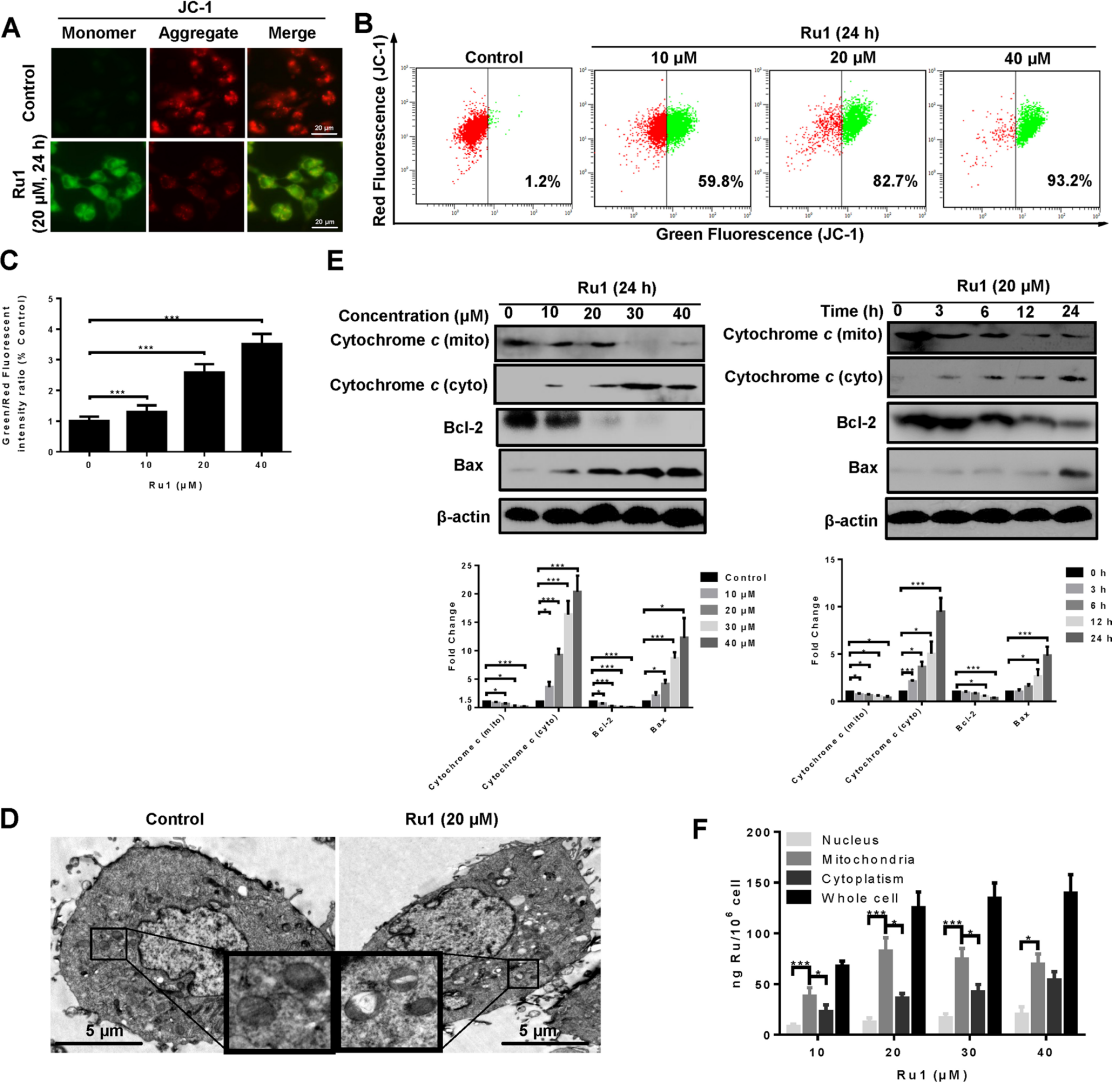
Ru1 induces mitochondrial dysfunction (**A**) Fluorescence microscope analysis of cellular MMP level by JC-1 staining after Ru1 treatment for 24 h. (**B** and **C**) Flow cytometry analysis of cellular MMP level after 10, 20 and 40 μM of Ru1 treatment for 24 h. (**D**) Two representative TEM images were identified to show the mitochondrial dysfunction after Ru1 treatment for 24 h. (**E**) The expression levels of cytochrome *c*, Bcl-2 and Bax were performed in a dose- and time-dependent manner. Below, quantification of the bands normalized by β-actin. (**F**) Ruthenium concentration in nucleus, mitochondria, cytoplasm, and whole cell (ng Ru/10^6^ cells) in A549 cells after 24 h of exposure to different concentrations of Ru1. Results were represented as mean ± SD (***p < 0.001).

Figure 2A and Supplementary Figure S2A showed representative images taken by using an inverted fluorescence microscope with Ru1 treatment. After Ru1 treatment of A549 and NCI-H460 cells, red fluorescence in cancer cells decreased and green fluorescence correspondingly increased, which indicated the decrease of MMP. Figure 2B and 2C displayed the decrease of MMP detected quantitatively by using flow cytometry. Additionally, transmission electron microscope (TEM) observations were also carried out to further confirm mitochondrial dysfunction [25, 26]. As shown in Figure 2D, swollen mitochondria appeared clearly after treatment, whereas the mitochondria in the control were normal. Meanwhile, western blot analysis indicated that treatment with Ru1 dose and time dependently suppressed the expression of Bcl-2 and increased the expression of Bax, thus leading to the release of mitochondrial proteins, such as cytochrome c. Actually, the experimental results suggested that cytochrome *c* of cytosol increased in a dose- and time-dependent manner, while those in mitochondria decreased (Figure 2E). These results demonstrated that Ru1 induced mitochondrial dysfunction. Similar results were also observed in Ru1 induced mitochondrial dysfunction in NCI-H460 cells (Supplementary Figure S2B).

To elucidate the underlying mechanisms of the mitochondrial dysfunction, Inductively Coupled Plasma Mass Spectrometry (ICP-MS) was used to detect the cellular-Ru1 distribution [27, 28]. As shown in Figure 2F, the absorbed dose increased after exposure to 10 and 20 μM of Ru1, and then remained at a steady state thereafter. In addition, Ru1 accumulation in mitochondria of A549 cells was higher than that in nucleus and cytoplasm, especially at lower doses. Moreover, the result further suggested that Ru1 may induce mitochondrial dysfunction directly.

### Ru1 stimulates ROS generation from mitochondria

**Figure 3:**
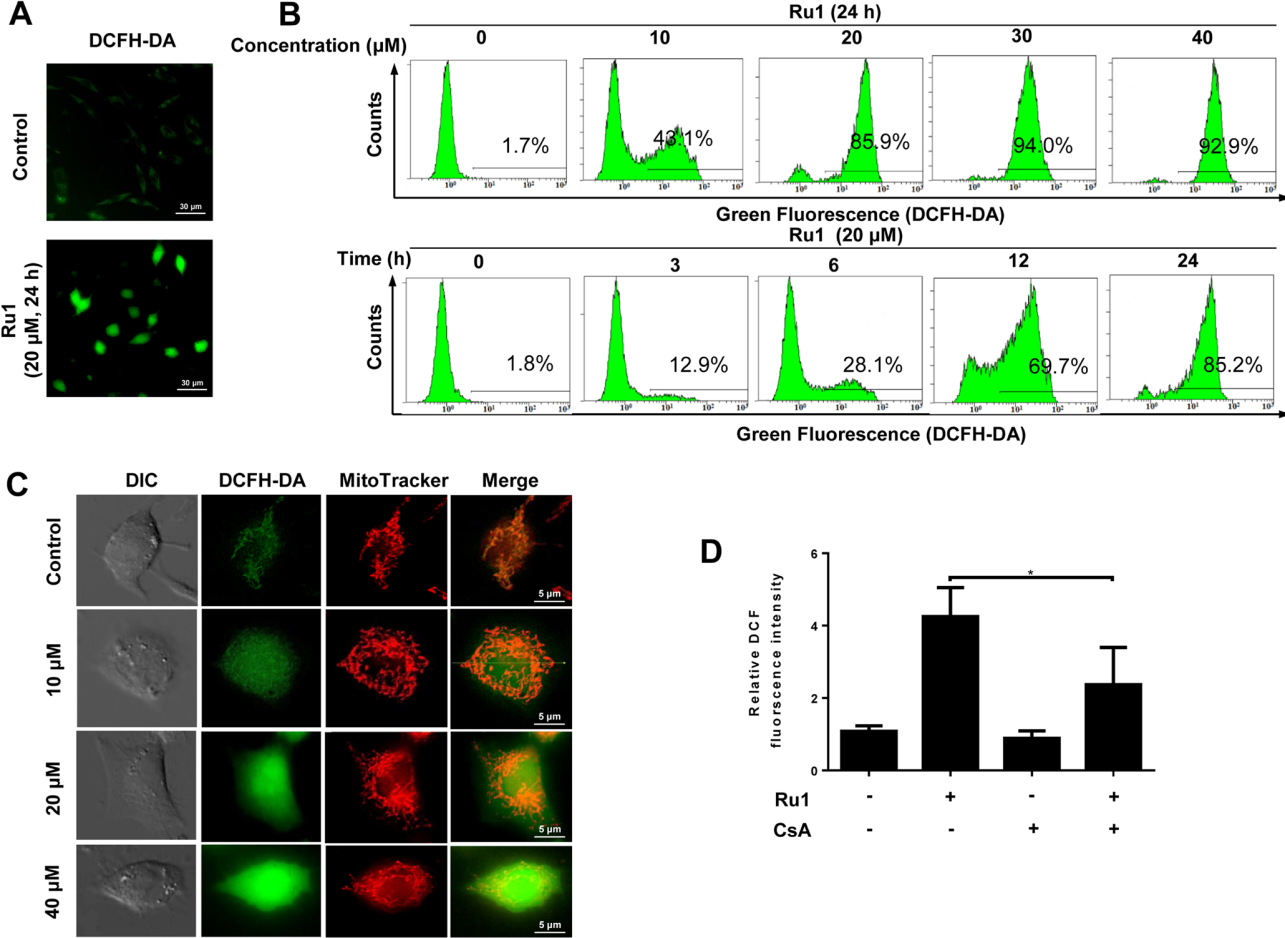
Ru1 stimulates generation of ROS from mitochondrial (**A**) Fluorescence microscope analysis of cellular ROS level by DCFH-DA staining after Ru1 treatment. (**B**) Flow cytometry analysis of cellular ROS level in a dose- and time-dependent manner after Ru1 treatment. (**C**) Ru1-triggered ROS co-localized with MitoTracker Red-stained mitochondria. Treated cells were stained with 20 nM of Mito Tracker-Red and 10 μM of DCFH-DA for 30 min. (**D**) Microplate analysis of cellular ROS level by DCFH-DA staining after 20 μM of Ru1 treatment for 24 h with, or without, 1-h CsA (2 μM) pre-treatment. Results were represented as mean ± SD (*p < 0.05).

ROS, which is considered to be a mediator of apoptosis, can trigger a series of mitochondria-associated events including MMP and apoptosis [6, 29, 30]. Hence, exploring the cellular ROS level is very important when elucidating the potential mechanisms triggered by Ru1. To determine whether, or not, Ru1 stimulates ROS generation, 2,7-dichlorodi-hydrofluorescein diacetate (DCFH-DA), a fluorescent dye, was used. Figure 3A and Supplementary Figure S2C suggested that, compared with DMSO-treated control, A549 and NCI-H460 cells-treated presented obvious green fluorescence, which manifested an increase in the cellular ROS level. As shown in Figure 3B, the results of detected quantitatively by using flow cytometry displayed there was a significant dose- and time-dependent increase in the cellular ROS level after Ru1 treatment, indicating that Ru1 could enhance ROS levels in A549 cells.

To determine whether, or not, Ru1-triggered ROS generated from mitochondria, the mitochondrial pattern of fluorophore 2,7-dichlorofluorescein (DCF) fluorescence was examined [31, 32]. Figure 3C and Supplementary Figure S2D showed that the DCF fluorescence partly co-localized with a mitochondrial marker, especially in control (or at low-dose Ru1 treatment). Moreover, it was found that cyclosporine A (CsA), which was an inhibitor of mitochondrial permeability transition pore (MPTP) opening, inhibited the Ru1-triggered ROS generation successfully (Figure 3D and Supplementary Figure S2E).

These results indicated that Ru1 could activate mitochondrial production of ROS, and further revealed that, the loss of MMP may be the main reason of ROS generation.

### Ru1-induced apoptosis is partially caspase 3-dependent and caused by triggering ROS-mediated mitochondrial dysfunction in A549 and NCI-H460 cells

**Figure 4:**
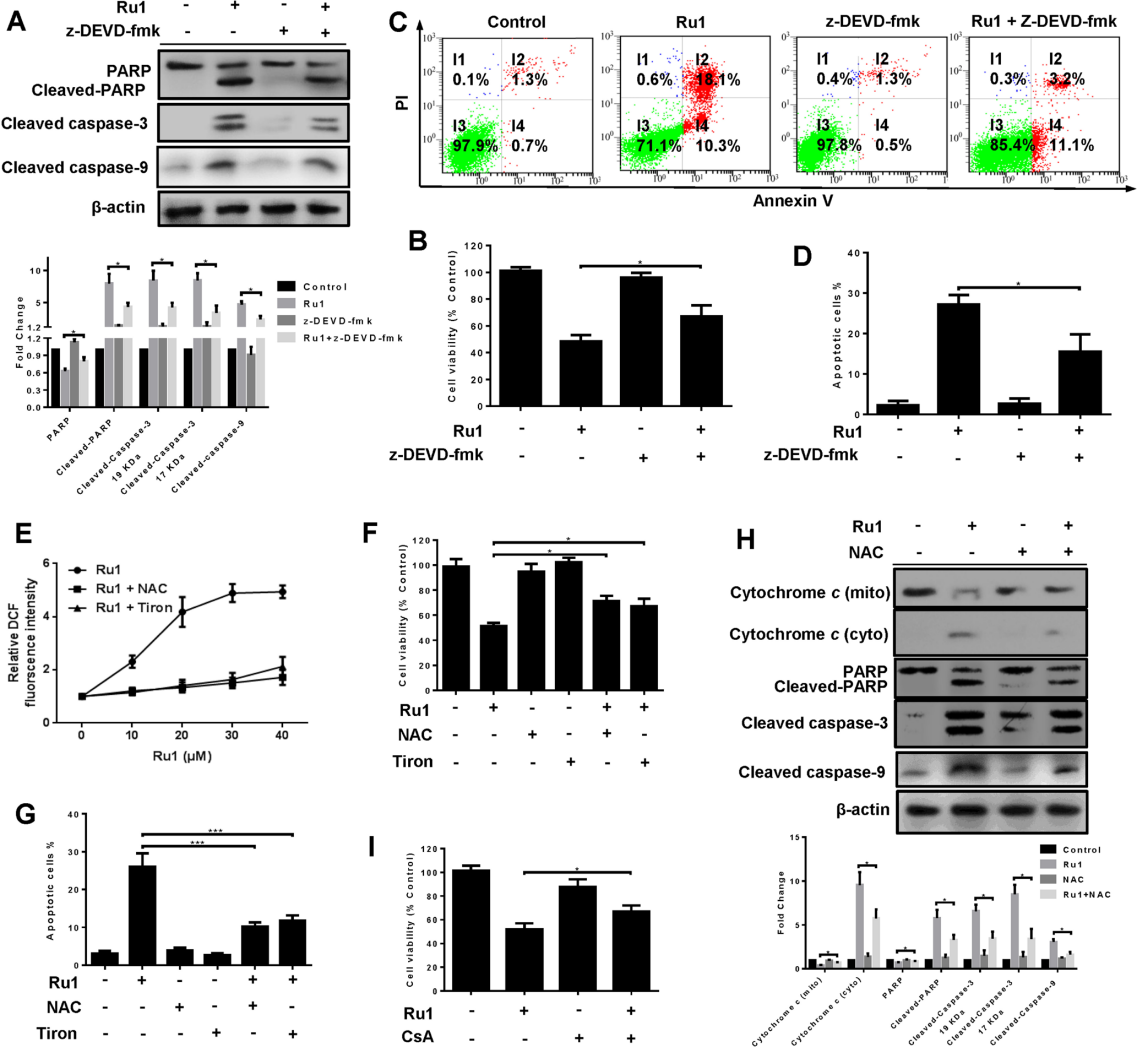
Ru1-induced apoptosis is partially caspase 3-dependent by triggering ROS-mediated mitochondrial dysfunction in A549 cells (**A**–**D**) A549 cells were incubated with Ru1 for 24 h with, or without, 1-h z-DEVD-fmk (50 μM) pretreatment. (A) The levels of cleaved caspase-3, PARP, cleaved-PARP and cleaved caspase-9 were assessed by western blot analysis. Below, quantification of the bands normalized by β-actin. (B) Cell viability was measured by MTT assay. (C and D) Annexin V/PI assay was performed by flow cytometry. (**E**–**H**) A549 cells were treated with Ru1 for 24 h with, or without, antioxidants (NAC = 10 mM and Tiron = 5 mM). (E) Relative fluorescence intensity was measured by microplate analyser. (F) Cell viability was assessed by MTT assay. (G) The percentage of apoptotic cells was measured by flow cytometry. (H) The levels of cytochrome *c*, cleaved-caspase 3, PARP, cleaved-PARP and cleaved-caspase 9 were assessed by western blot analysis with, or without, antioxidants (NAC = 10 mM). Below, quantification of the bands normalized by β-actin. (**I**) Cell viability was assessed by MTT assay after Ru1 treatment for 24 h with, or without, 1-h CsA (2 μM) pre-treatment. Results were represented as mean ± SD (*p < 0.05, ***p < 0.001).

A caspase 3 inhibitor – z-DEVD-fmk was used to further demonstrate whether, or not, Ru1-induced apoptosis in A549 and NCI-H460 cells was correlated to the activation of caspase 3. The results manifested that z-DEVD-fmk could significantly decrease the level of cleaved-PARP, cleaved caspase-3 and cleaved caspase-9 (Figure 4A and Supplementary Figure S3A). In addition, pre-treatment with z-DEVD-fmk (50 μM) increased the percentage of cell viability (Figure 4B and Supplementary Figure S3B) and decreased the percentage of apoptotic cells (Figure 4C, 4D and Supplementary Figure S3C, S3D).

Two antioxidants, *N*-acetylcysteine (NAC, 10 mM) and 4,5-dihydroxy-1,3-benzenedisulphonic acid disodium salt (Tiron, 5 mM), were used to investigate the roles of ROS induced by Ru1. As shown in Figure 4E and 4F, antioxidants suppressed Ru1-induced ROS generation and increased cell viability. When antioxidants (NAC or Tiron) were present, all the percentage of apoptotic cells (Figure 4G), the release of cytochrome *c* in mitochondria, and the level of cleaved-PARP, cleaved caspase-3 and cleaved caspase-9 (Figure 4H and Supplementary Figure S3E) significantly decreased. In addition, pre-treatment with CsA effectively prevented the Ru1-triggered cell growth inhibition, and the percentage of cell viability increased (Figure 4I).

These findings revealed that the apoptosis-induced by Ru1 in A549 and NCI-H460 cells was partially caspase 3-dependent by triggering ROS-mediated mitochondrial dysfunction.

### Ru1 induces autophagy and autophagic flux in A549 and NCI-H460 cells

**Figure 5:**
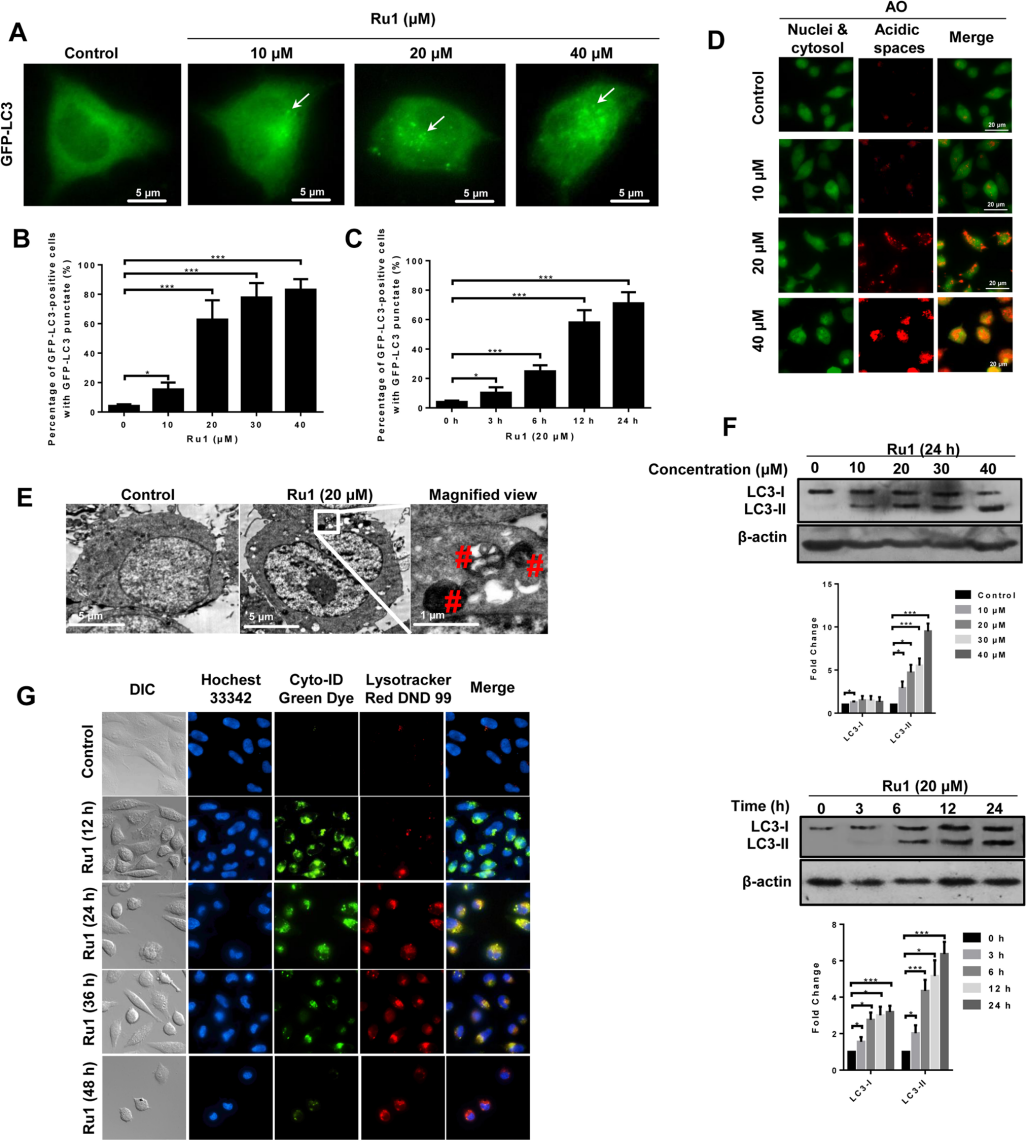
Ru1 induces autophagy and autophagic flux in A549 cells (**A**) Fluorescence microscope analysis of autophagy in A549 cells stained with GFP-LC3 after Ru1 treatment for 24 h. (**B** and **C**) GFP-LC3-postive cells were quantified in a dose- and time-dependent manner during Ru1 treatment. Data are presented as percentages of GFP-LC3-postive cells with GFP-LC3 punctate fluorescence. (**D**) Fluorescence microscope analysis of cellular AVOs level by AO staining after different concentrations Ru1 treatments for 24 h. (**E**) TEM images showing the ultrastructure of A549 cells treated with 20 μM of Ru1 for 24 h. (**F**) Western blot analysis of the levesl of LC3-I and LC3-II in dose- and time-dependent Ru1 treatment. Below, quantification of the bands normalized by β-actin. (**G**) Autophagic flux induced by Ru1. Cells were co-stained with Cyto-ID green dye and lysotracker red DND 99. Results were represented as mean ± SD (***p < 0.001).

Previous research has reported that some Ru(II)-*β*-carboline complexes could induce autophagy in cancer cells [9]. Here, we investigated whether, or not, autophagy was also involved in the process of Ru1-induced cell death. The conversion of the cytosolic LC3-I to the autophagosome-associated LC3-II form is considered as a hallmark of autophagy [33, 34]. To determine whether, or not, LC3 was concentrated in A549 and NCI-H460 cells after Ru1 treatment, GFP-LC3 transfection of A549 and NCI-H460 cells was performed, and the presence of a bright green fluorescence with a punctate pattern of GFP-LC3 expression (GFP-LC3 dots) was examined by using fluorescence microscopy. Figure 5A and Supplementary Figure S4A displayed that, in control cells, GFP-LC3 protein was diffusely distributed throughout the cytoplasm, indicating the absence of classical autophagy in cells. When A549 or NCI-H460 cells were pre-incubated with Ru1, the number and frequency of GFP-LC3 dots were significantly increased. The percentage undergoing autophagy was quantified by counting the number of GFP-LC3-positive cells (≥ 5 GFP-LC3 dots per cell) in 300 GFP-LC3-transfected cells. Figure 5B, 5C and Supplementary Figure S4B showed that GFP-LC3-positive cells increased in a concentration- and time-dependent manner after Ru1 treatment.

Autophagy is characterised by the formation and promotion of AVOs [35]. To detect the AVOs in Ru1-treated cells, we performed cell staining with acridine orange (AO), which can emit weak red fluorescence in acidic spaces in the cytoplasm [36, 37]. The intensity of the red fluorescence is proportional to the degree of acidity. As shown in Figure 5D and Supplementary Figure S4C, A549 and NCI-H460 cells without Ru1 treatment exhibited green fluorescence (control), whereas cells treated with Ru1 showed an increase in red fluorescence in a dose-dependent manner: this revealed an increase in the number of cytoplasmic AVOs, a characteristic of autophagy.

TEM is considered as the most convincing means used in the study of autophagy because its high resolution makes it capable of observing the ultrastructure of cells [33, 38]. Representative TEM images are shown in Figure 5E and Supplementary Figure S4D. Compared with the few autolysosomes observed in control cells, typical autolysosomes were observed in A549 and NCI-H460 cells after Ru1 treatment.

In addition to fluorescence microscopy and TEM observation, LC3 immunoblotting is another common method used to detect LC3 conversion. When autophagy occurs, the soluble form of LC3 (LC3-I) is converted into the liposoluble form (LC3-II), and the amount of which is closely related to the number of autophagosomes [39]. Figure 5F and Supplementary Figure S4E showed that Ru1 treatment significantly enhanced the expression of LC3-II protein in a concentration- and time-dependent manner.

To further evaluate the role of autophagy in Ru1-induced apoptosis, we detected if autophagic flux was induced after lung cancer cells were exposed to Ru1. The autophagic flux was determined by using Cyto-ID and LysoTracker staining as reported [40, 41]. As shown in Figure 5G, Ru1 induced formation and accumulation of autophagosomes (green fluorescence), fusion of autophagosomes with lysosomes (yellow fluorescence) and clearance of autophagosomes by lysosomes (red fluorescence) in a time-dependent manner in A549 cells. Similar results were also observed in Ru1-treated NCI-H460 cells (Supplementary Figure S4F). Thus, our results indicated that autophagy and autophagic flux were induced in Ru1-treated A549 and NCI-H460 cells.

### Inhibition of autophagy enhances Ru1-induced growth inhibition and apoptosis of A549 and NCI-H460 cells

Several researches have suggested that autophagy induced by antitumor compounds may either protect cancer cells from cell death or contribute to cancer cell death [42, 43]. To explore the role of Ru1-induced autophagy in apoptosis, autophagy inhibitors 3-MA and CQ were used to block autophagy in A549 and NCI-H460 cells, and the effects on the level of LC3-II and Ru1-induced cell-viability, as well as apoptosis, were detected by western blot analysis, MTT assay and flow cytometry [44]. 3-MA is an inhibitor of class III phosphoinositide 3-kinases (PI3Ks), which inhibits the conversion of LC3-I to LC3-II by inhibiting the activity of PI3Ks. However, CQ, a lysosome inhibitor, can enhance LC3-II levels by interfering with the acid-dependent degradation of proteins within the autophagosome.

**Figure 6:**
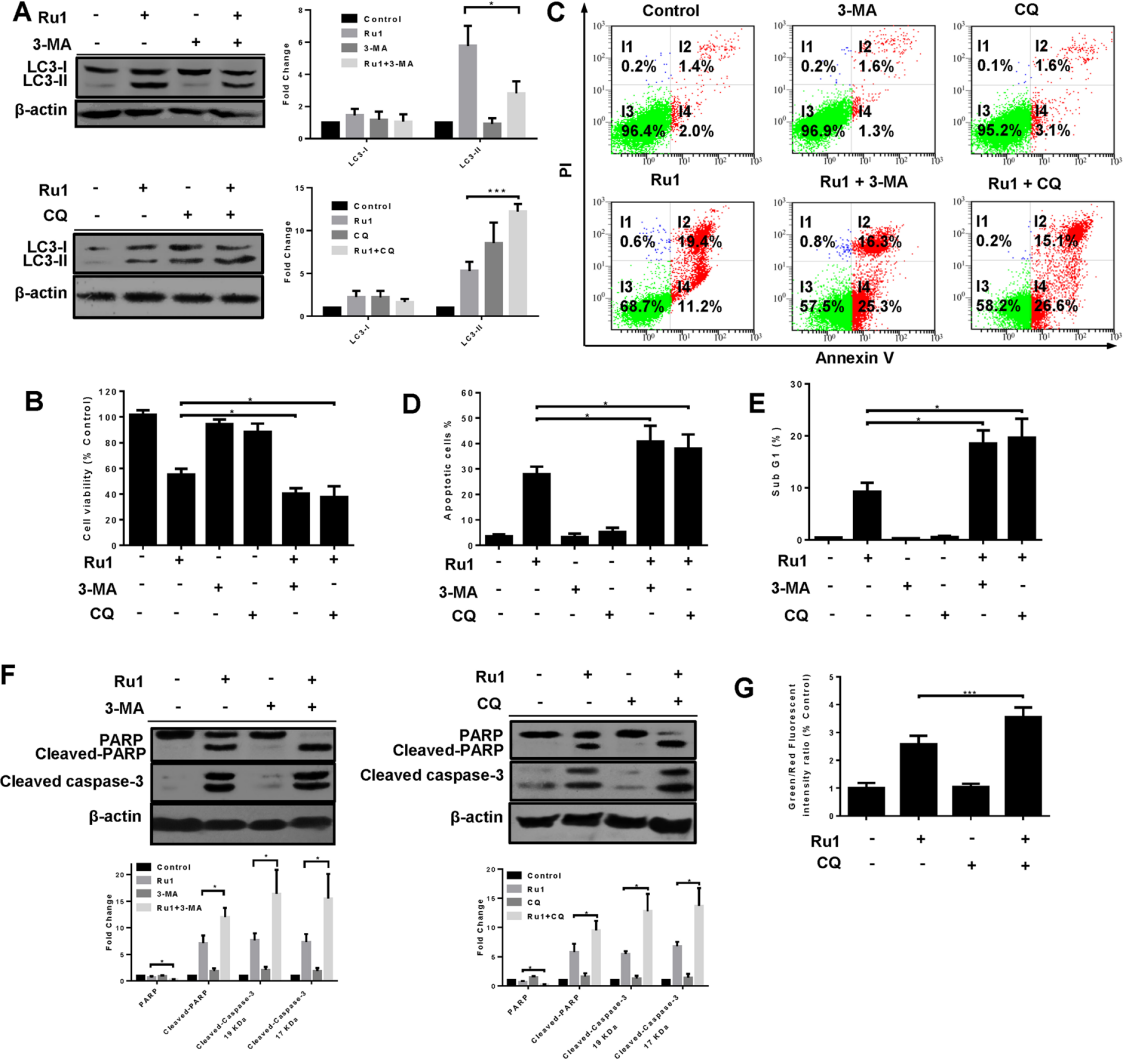
Inhibition of autophagy enhances Ru1-induced growth inhibition and apoptosis of A549 cells (**A**–**G**) Cells were pre-incubated with 10 mM 3-MA or 2.5 μM CQ for 1 h, and then exposed to Ru1 for 24 h. (A) Autophagy-associated protein LC3-I/II was detected by western blot analysis. Right, quantification of the bands normalized by β-actin. The percentage of cell viability (B), apoptosis cells (C and D), and sub-G1 (E) were obtained by MTT assay and flow cytometry. (F) The levels of cleaved-caspase 3, PARP, and cleaved-PARP were assessed by western blot analysis. Below, quantification of the bands normalized by β-actin. (G) Flow cytometry analysis of cellular MMP level by JC-1 staining. Results were represented as mean ± SD (*p < 0.05).

As shown in Figure 6A and Supplementary Figure S5A, western blot analysis manifested that 3-MA and CQ obviously inhibited Ru1-induced autophagy. In additional, compared with A549 or NCI-H460 cells that were exposed to Ru1, treatment with 3-MA or CQ significantly decreased cell viability (Figure 6B and Supplementary Figure S5B). To further understand the function of autophagy in Ru1-induced cell death, the changes in Ru1-induced apoptosis was examined. These findings demonstrated that Ru1, in combination with 3-MA or CQ, induced a higher percentage of apoptotic cells (Figure 6C–6E and Supplementary Figure S5C–5E) and more cleaved-PARP and cleaved caspase-3 (Figure 6F and Supplementary Figure S5F) when compared with Ru1-treatment alone. Besides, the lung cells incubated with 3-MA or CQ alone showed limited apoptosis-inducing effects on A549 or NCI-H460 cells. All of these findings implied that inhibition of autophagy increased Ru1-induced cell death.

Past research has shown that the activation of caspases is driven by cytochrome *c* via an intrinsic mitochondrial apoptotic pathway [45–47]. Hence, we investigated the impact of inhibited autophagy on the mitochondria using flow cytometry by JC-1 staining. Figure 6G showed that inhibition of autophagy in Ru1-treated A549 cells induced an increase in the percentage of green fluorescence, indicating a loss of more MMP. These data suggested that autophagy reduced Ru1-induced growth inhibition and apoptosis of A549 and NCI-H460 cells may arise from the elimination of that portion of the damaged mitochondria.

### The ERK signaling pathway is involved in ROS-dependent autophagy triggered by Ru1 in A549 and NCI-H460 cells

**Figure 7:**
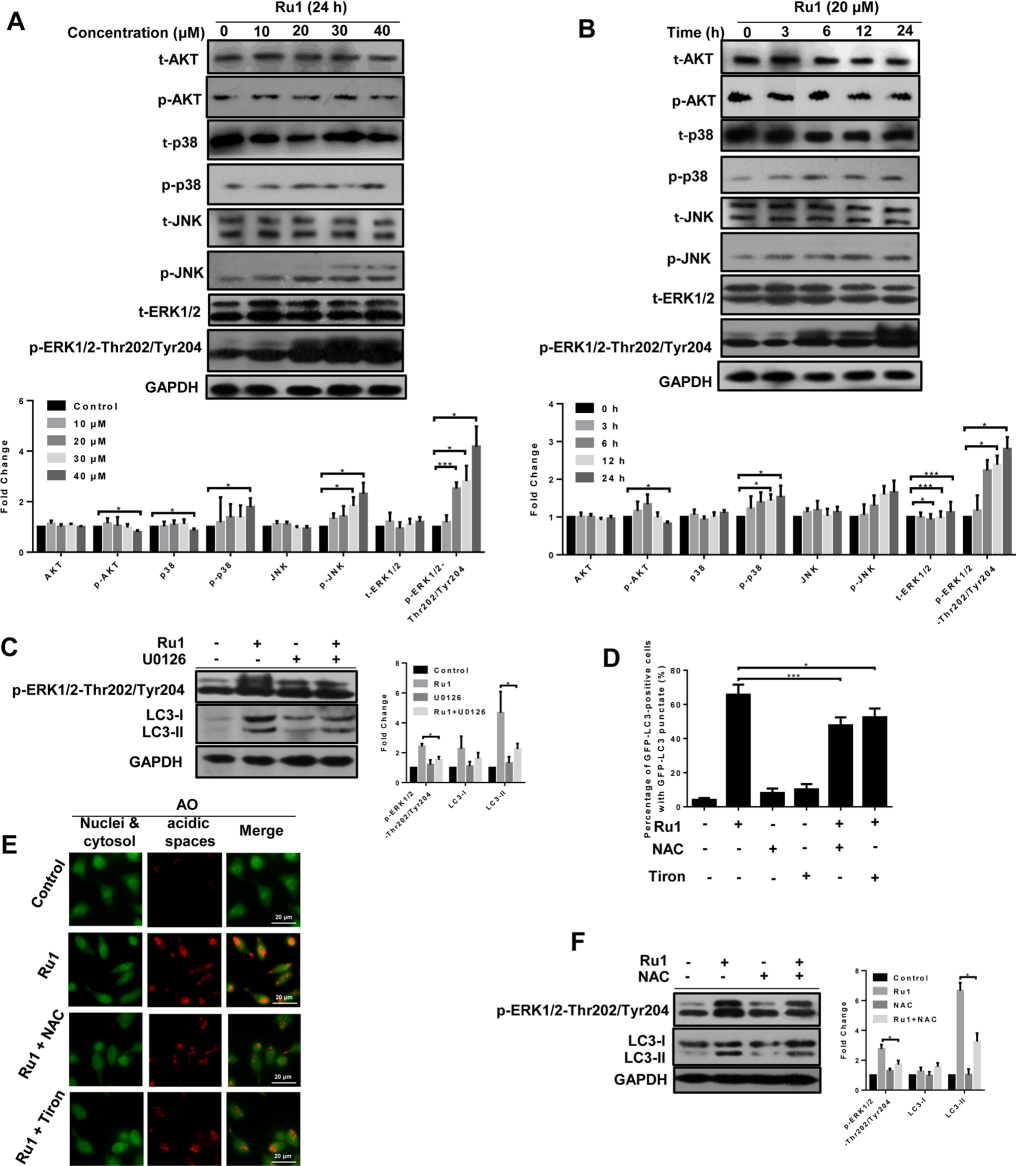
The ERK signaling pathway is involved in ROS-dependent autophagy induced by Ru1 in A549 cells (**A** and **B**) Western blot analysis of the expression level of total and the phosphorylation status of MAP kinases, AKT and ERK1/2. Below, quantification of the bands normalized by GAPDH. (**C**) Western blot analysis of the levels of LC3-I/II and p-ERK1/2. Right, quantification of the bands normalized by GAPDH. A549 cells were incubated with 20 μM of Ru1 for 24 h with, or without, the ERK phosphorylation inhibitor U0126 (20 μM) pre-treatment. (**D**) GFP-LC3-postive cells were quantified after 20 μM of Ru1 treatment for 24 h with, or without, 1-h NAC (10 mM) or Tiron (5 mM) pre-treatment. (**E**) Fluorescence microscope analysis of cellular AVOs level by AO staining after 20 μM of Ru1 treatment for 24 h with, or without, 1-h NAC (10 mM) or Tiron (5 mM) pre-treatment. (**F**) Western blot analysis of the level of LC3-I/II and p-ERK1/2. Right, quantification of the bands normalized by GAPDH. A549 cells were incubated with 20 μM of Ru1 for 24 h with, or without, 1-h NAC (10 mM) pre-treatment. Results were represented as mean ± SD (*p < 0.05, ***p < 0.001).

Mitogen-activated protein kinases (MAPKs, including ERK1/2, JNK and p38 MAPK), and PI3K/Akt signaling pathways, play vital roles in regulating cellular processes via downstream signal transduction cascades [48, 49]. To determine whether, or not, these pathways were involved in the Ru1-induced autophagy, the expressions of total and phosphorylated AKT, p38, JNK and ERK1/2 were detected by western blot analysis. After Ru1 treatment, the level of phospho-ERK1/2 (p-ERK1/2) increased dramatically in a concentration- and time-dependent manner, whereas no significant changes occurred in the level of p-JNK, p-p38, p-AKT and total protein expression (Figure 7A, 7B and Supplementary Figure S6A, S6B). p-ERK1/2 has been reported as an inducer of autophagy by interacting with LC3 [50]. To further inquire into the role of p-ERK1/2 in Ru1-induced autophagy, U0126 (an ERK1/2 phosphorylation inhibitor) was used to block p-ERK1/2. As shown in Figure 7C and Supplementary Figure S6C, the expression of LC3-II and p-ERK1/2-Thr202/Tyr204 were decreased in A549 or NCI-H460 cells after treatment with Ru1 and U0126 for 24 h, suggesting that autophagy was suppressed by inhibiting the phosphorylation of ERK1/2.

Early reports have demonstrated that ROS plays a vital role in the induction of autophagy [51–53]. In order to investigate the relationship between cellular ROS levels and autophagy, we used antioxidants NAC and Tiron to inhibit ROS levels. As shown in Figure 7D, 7E and Supplementary Figure S6D, S6E, in the presence of NAC or Tiron, the percentage of GFP-LC3 positive cells and cellular AVOs level (character by the intensity of red fluorescence) decreased after Ru1 treatment, suggesting that autophagy was suppressed by inhibiting cellular ROS levels. Meanwhile, Figure 7F and Supplementary Figure S6F showed that the levels of LC3-II and p-ERK1/2-Thr202/Tyr204 decreased in A549 and NCI-H460 cells after exposure to Ru1 for 24 h with NAC pre-treatment, indicating that an ERK signaling pathway was involved in ROS-dependent autophagy induced by Ru1 in A549 and NCI-H460 cells.

### Ru1 inhibits growth of A549 cells xenografts *in vivo*

**Figure 8:**
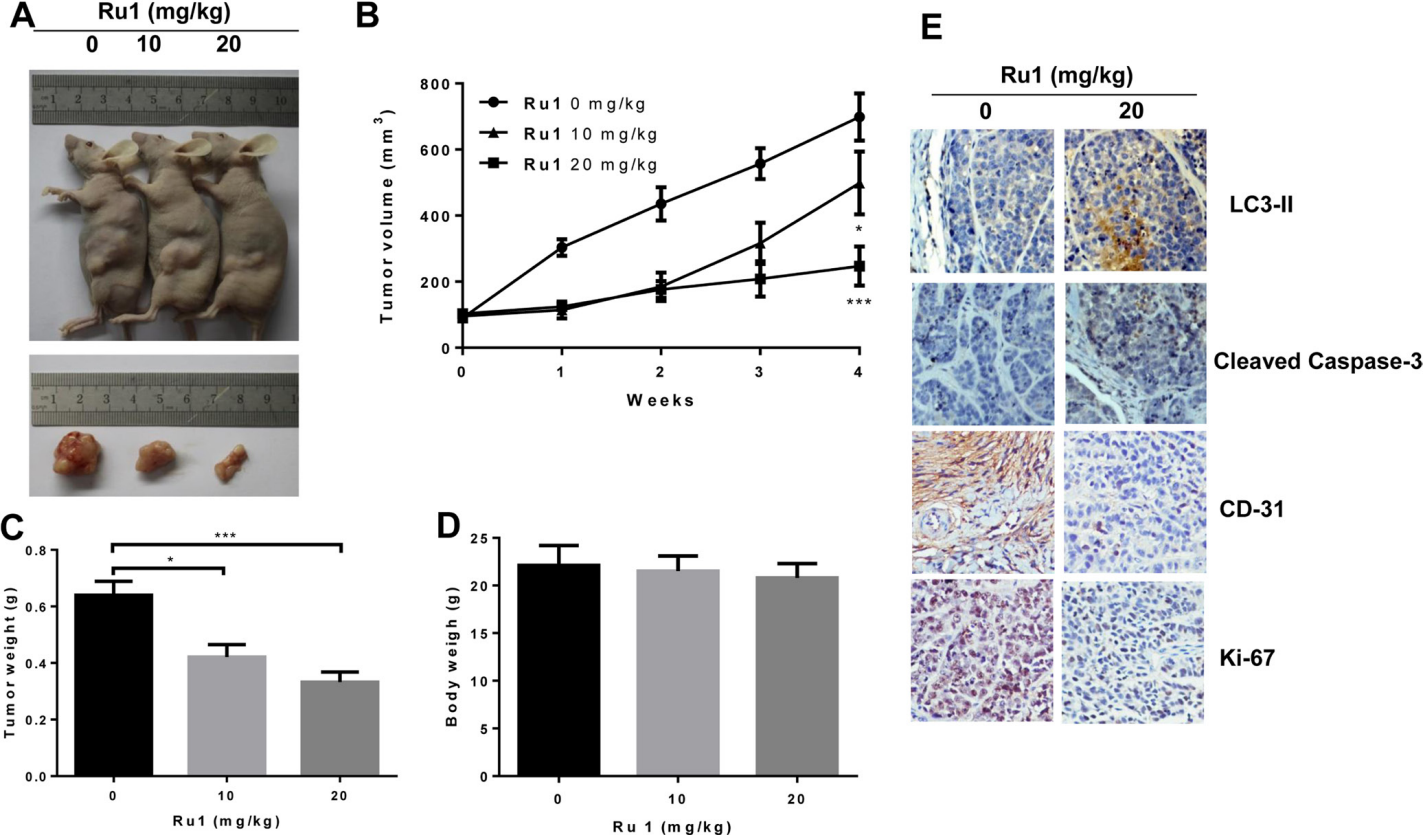
Ru1 inhibites the tumor progression of lung cancer *in vivo* (**A**) Athymic nude mice injected (s.c.) with A549 cells were injected with Ru1 (0, 10 or 20 mg/kg, i.p.). (**B**) The volume of tumors was monitored in terms of tumor volume every week. The weights of the mice tumors (**C**) and the body (**D**) were determined, (**E**) Immunohistochemical analysis of LC3-II, cleaved caspase-3, CD-31 and Ki-67 expression in tumor tissue. All images were representative and were taken at 200× magnification. Results were represented as mean ± SD (*p < 0.05, ***p < 0.001, compared with that of the untreated control).

To evaluate whether Ru1 could inhibit the progression of lung cancer cells *in vivo*, we performed an antitumor study using athymic nude mice injected (s.c.) with A549 cells. After the administration of Ru1 at 10 and 20 mg/kg/3 day for 28 days, the weight and volume of tumors were significantly reduced (Figure 8A, 8B, and 8C), compared with the control group. In addition, the mean body weight of nude mice with A549 tumor xenografts was no significant differences (Figure 8D). Immunohistochemical (IHC) staining results (Figure 8E) revealed that LC3-II and cleaved caspase-3 were up-regulated by Ru1 treatment, which was consistent with the *in vitro* results. Furthermore, CD-31 and Ki-67 expression demonstrated that Ru1 could inhibit angiogenesis and cell proliferation in tumor xenografts. Taken together, these results suggested that Ru1 could induce autophagy, apoptosis and inhibits tumor growth *in vivo*.

## DISCUSSION

In this study, we observed that a ruthenium (II) imidazole complex [Ru(Im)_4_(dppz)]^2+^ (Ru1) could induce growth inhibition and apoptosis in A549 and NCI-H460 cells (Figure 1 and Supplementary Figure S1). Further studies illustrated that Ru1-induced apoptosis was partially caspase 3-dependent in A549 and NCI-H460 cells. Moreover, LC3-II, a marker of autophagy, was obviously increased after Ru1 treatment in A549 and NCI-H460 cells (Figure 5F and Supplementary Figure S4E). Besides, the increasing formation of autophagosomes and autolysosomes over time, as detected by TEM (Figure 5E and Supplementary Figure S4D), and the increased formation of characteristic AVOs and GFP-LC3 expression in cytoplasm provided another two pieces of evidence of this (Figure 5A–5D and Supplementary Figure S4A–4C). All of these findings suggested that Ru1 could trigger autophagy.

More and more evidence suggests that mitochondria and ROS are important in the process of apoptosis-inducing in many cancer cells [54, 55]. Therefore, we investigated whether, or not, Ru1 treatment could induce mitochondrial dysfunction and enhance ROS level in A549 and NCI-H460 cells. As an indispensable organelle for energy production in eukaryotic cells, mitochondria are crucial regulators of the intrinsic pathway of apoptosis [56]. In our study, the loss of MMP and release of cytochrome *c* indicated that Ru1 could induce mitochondrial dysfunction in a concentration- and time-dependent manner (Figure 2A–2E and Supplementary Figure S2A, S2B). Moreover, the mitochondrial accumulation of Ru1 further informed us that Ru1 may induce a distinct loss of MMP directly (Figure 2F). Meanwhile, the apparent loss of MMP is often accompanied by increasing ROS generation, which, in turn, leads to rapid saturation of the antioxidant systems and induces the functional impairment of mitochondria [57]. That is also confirmed in Ru1 treatment: Ru1 targeted mitochondria, and then triggered the loss of MMP accompanied by the increased generation of ROS that eventually facilitated the permeabilization of the mitochondrial outer membrane, followed by the release of cytochrome *c*, which subsequently stimulated greater ROS formation. When inhibited by mitochondrial permeability transition pore (MPTP) opening, Ru1-triggered ROS generation was successfully suppressed (Figure 3D and Supplementary Figure S2E). These results demonstrated that the loss of MMP was the major cause of ROS generation.

As a vital mediator of apoptosis, the role of ROS is becoming increasingly recognised. To inquire into the contribution of ROS in Ru1-induced A549 and NCI-H460 cells apoptosis, we used antioxidants (NAC or Tiron) and CsA in this study. As expected, scavenging of ROS inhibited the release of cytochrome *c*, the activation of cleaved caspase-3, cleaved caspase-9, and Ru1-induced apoptosis (Figure 4E–4H, Supplementary Figure S3E). In addition, inhibition of MPTP opening increased the cell viability (Figure 4I). Our studies implied that the ROS accumulation enhanced caspase 3-dependent apoptosis in A549 and NCI-H460 cells. The findings further indicated that ROS-mediated mitochondrial dysfunction triggered Ru1-induced apoptosis in A549 and NCI-H460 cells.

In cancer chemotherapy, autophagy can either promote cell death, or protect cancer cells from cell death, remains a controversial matter [17, 18, 21, 58]. Although previous research reports that some ruthenium β-carboline complexes induce autophagy in cell death, the role of autophagy in ruthenium complex-induced cell death is still unclear [9]. In our studies, the autophagy inhibitors both 3-MA and CQ can inhibit Ru1-induced autophagy (Figure 6A and Supplementary Figure S5A). Remarkably, Ru1 and autophagy inhibitors treatments decreased cell viability in A549 and NCI-H460 cells (Figure 6B and Supplementary Figure S5B), and enhanced Ru1-induced apoptosis as evinced by the increased percentage of apoptotic cells, the level of cleaved caspase-3 and cleaved-PARP (Figure 6C–6F and Supplementary Figure S5C–5F). All of these findings demonstrated that Ru1-induced autophagy played a cytoprotective role in A549 and NCI-H460 cells. Blocking autophagy could enhance the efficacy of Ru1 on A549 and NCI-H460 cells, and that might be a promising new therapeutic strategy for lung cancer. Furthermore, our results indicated that autophagy inhibition in Ru1-treated A549 cells triggered the loss of more MMP (Figure 6G), suggesting that autophagy antagonized Ru1-induced apoptosis of A549 cells perhaps through eliminating the damaged portion of the mitochondria.

Activation or inhibition of ERK signaling pathway plays a vital role in regulating cellular apoptosis and autophagy. However, to our knowledge, the ERK-dependent autophagy triggered by ruthenium complexes has not yet been reported. There are many reports of other drugs or materials: Sivaprasad *et al.* have reported that tumour necrosis factor induced autophagy through an ERK1/2 signaling pathway [59]. Gong *et al.* point out that the ERK signaling pathway at least partially facilitated tetrandrine-induced autophagy in hepatocellular cancer cells [55]. In addition, it was reported that the ERK1/2 signaling pathway was involved in autophagy induced by a combination of treatments involving arsenic trioxide and irradiation [60]. Our results suggested that, upon Ru1 treatment, the expression levels of p-ERK1/2 increased dramatically in a dose- and time-dependent manner, whereas no significant changes took place in the levels of p-JNK, p-p38, p-AKT, t-ERK1/2, t-JNK, t-p38 and t-AKT (Figure 7A, 7B and Supplementary Figures S6A, S6B). Meanwhile, inhibition of p-ERK1/2 attenuated the Ru1-induced autophagy (Figure 7C and Supplementary Figure S6C). These findings suggested that an ERK signaling pathway was involved in Ru1-induced autophagy in A549 and NCI-H460 cells.

Early reports point out that ROS accumulation could promote the level of ERK and regulate autophagy [55, 61]. Therefore, it is necessary to investigate the role of ROS in Ru1-induced cells autophagy. As expected, our findings demonstrated that blocking of ROS accumulation with antioxidant NAC and Tiron substantially inhibited the Ru1-induced A549 and NCI-H460 cells autophagy (Figure 7D–7F and Supplementary Figure S6D–6F), indicating that ROS accumulation is an important mechanism in the sensitisation of cells to apoptosis under autophagy inhibition conditions. These results were in accordance with early studies of 5-FU and cisplatin in which it was found suppressed autophagy could enhance the efficiency of chemotherapy by ROS accumulation [62, 63].

The role of autophagy in platinum-based therapies has been evaluated previously to probe and attempt to solve drug-resistance [63–67]. With respect to the ruthenium-based drugs, the research of autophagy is relatively scarce [9, 68]. Ruthenium-based drugs are very absorbing to study, not only to have certain advantages over platinum-based drugs, such as lower toxicity toward normal tissue, but also because they show different modes of action compared with platinum-based drugs [1]. Therefore, elucidating the underlying mechanisms, such as apoptosis and autophagy induced by ruthenium-based drugs is a very important work. Our limited studies in this work may lead to the development of ruthenium (II) imidazole complex in combination with autophagy inhibitors as a potential therapy regime for lung cancer therapy.

**Scheme 1:**
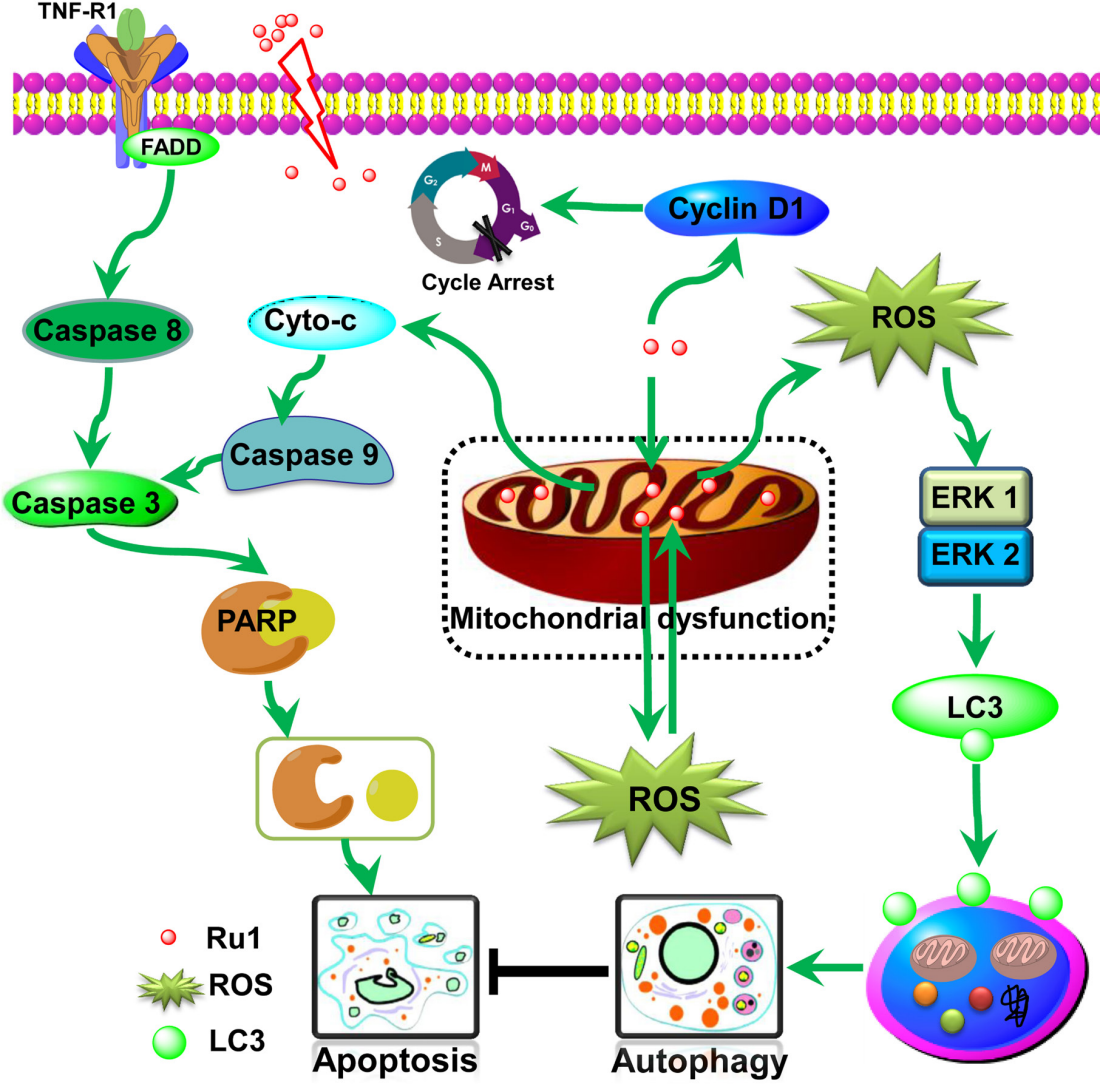
Proposed apoptosis and autophagy pathways induced by Ru1 in A549 cells Ru1 causes mitochondrial dysfunction, along with ROS generation from mitochondria, and thus induces partially caspase 3-dependent apoptosis and partially ERK-mediated autophagy in A549 cells. Moreover, the accumulation of Ru1 in mitochondria induces autophagy to antagonise mitochondrial-mediated apoptosis.

In conclusion, our studies show that the ruthenium imidazole complex Ru1 could induce G0/G1 arrest and cell apoptosis in A549 and NCI-H460 cells through extrinsic and intrinsic mitochondrial pathways. Since the mitochondrial membrane has negative charge, and Ru1 is a cationic compound with positive charge, the higher mitochondrial membrane potential in tumor cells may provide an opportunity for Ru1 to enter into mitochondria more faster, which lead to the decline of membrane potential and mitochondrial dysfunction, and then stimulation of ROS generation. ROS, generates from the mitochondria, participates in and regulates the mitochondria mediated apoptosis pathway, that is, the production and aggregation of ROS final accelerates the depolarization of the outer membrane of the mitochondria, which subsequently enhances Ru1-induced apoptosis in lung cancer cells. Further study shows that Ru1 could induce autophagy, whereas inhibition of autophagy could induce the reduction of mitochondrial membrane potential and facilitate cell apoptosis. Meanwhile, Ru1-induced cytoprotective autophagy mainly via ROS-mediated ERK signal pathway. The schematic diagram of this proposed apoptosis and autophagy pathway induced by Ru1 in A549 and NCI-H460 cells is illustrated in Scheme 1. As far as we know, it is the first report of a ruthenium imidazole complex which induced both apoptosis and autophagy in A549 and NCI-H460 cells as well as the first exposition of the underlying related behaviour mechanisms.

## MATERIALS AND METHODS

### Materials and cell culture

Ru1 and Ru2 were prepared according to the literature methods [13]. Cisplatin was purchased from Acros. Annexin V-FITC Apoptosis Detection Kit, Cell Mitochondria Isolation Kit, QuantiProTM BCA Assay Kit, ECL^™^ Start Western Blotting Detection Reagent, DMSO, MTT, phosphate buffered saline solution (PBS), PI, JC-1, NAC, Trion, DCFH-DA, CQ, 3-MA and z-DEVD-fmk were purchased from Sigma-Aldrich (St. Louis, MO, USA). CsA was purchased from Beyotime (Nantong, China). MitoTracker Red was purchased from Invitrogen. MAPK Family Antibody Sampler Kit (#9926, including ERK1/2, p38 MAPK, JNK, and secondary antibody), Phospho-MAPK Family Antibody Sampler Kit (#9910, including p-ERK1/2, p-p38 MAPK, p-JNK, U0126, and secondary antibody), LC3A/B (#12741), Cyclin D1 (#2978), PARP (#9542), Cytochrome *c* (#11940), Bax (#5023), Bcl-2 (#2870), β-actin (#4970), GAPDH (#2118), AKT (#4691), Phospho-AKT (#4060), Cleaved-Caspase 8 (#9496), Cleaved-Caspase 9 (#7237), Cleaved-Caspase 3 (#9661), Skp2 (#4358), p21 (#2947), p27 (#3686), and secondary antibody (#7074) were purchased form Cell Signaling Technology Company. Four different tumor cell lines A549 (CCL-185), HeLa (CCL-2), MCF-7 (HTB-22), HepG-2 (HB-8065) and NCI-H460 (HTB-177) and one normal cell line (HBE, CRL-2741) were purchased from American Type Culture Collection (ATCC, Manassas, VA). All cell lines were cultured in either Roswell Park Memorial Institute 1640 (RPMI 1640) culture media supplemented with 10% fetal bovine serum (FBS) and incubated in incubator with 5% CO_2_ at 37°C unless otherwise noted. Protein bands were visualized by using ChemiDocTM XRS+ Imaging System (Bio-Rad, USA). Flow cytometry was performed by EPICS XL-MCL (BECKMAN COULTER, USA) and microscopic observation was performed by Ti-E (Nikon, Japan).

### *In vitro* cytotoxicity assay

Four cancer cell lines and one normal cell were used to determine the biological activity of Ru1 and Ru2 by MTT assay. Cells were placed in 96-well plates at density of 5 × 10^3^ cells per well and grown overnight in incubator. Ru(II)-complexes were added in a time- and dose-dependently methods (DMSO for negative control). After incubation, MTT was then added to each 96-well plates for 4 h. The suspension was replaced with DMSO (150 μL/well) to dissolve the fresh formazan at room temperature. The optical density of each well was then measured with a microplate analyzer (Fluoroskan Ascent FL, Thermo, USA) at the wavelength of 570 nm. The IC_50_ values were obtained from the analysis of absorbance data.

### Apoptosis assay by Annexin V/PI double staining

Annexin V-FITC Apoptosis Detection Kit was used to perform the assay. After incubation with Ru1 for 24 h, cells were harvested and washed twice with PBS, and then resuspended in 500 μL binding buffer. The suspension was stained with 5 μL Annexin V-FITC and 10 μL PI at room temperature for 15 min in the dark, and then analyzed using the flow cytometer. Another apoptosis assay stained Annexin V alone was also performed with the identical method. When necessary, 2.5 μM CQ, 10 mM 3-MA, or 50 μM z-DEVD-fmk was added firstly in medium for 1 h before Ru1 treatment.

### Western blotting analysis

For total protein isolation, cells were harvested in lysis buffer containing radio immunoprecipitation assay (RIPA) buffer, PMSF (phenylmethanesulfonyl fluoride) and phosphatase inhibitors mixture. For cytosolic protein (cytochrome *c*), the cytosol fraction was isolated from the total cell lysates using Cell Mitochondria Isolation Kit (brief method provided in the following part). QuantiPro^™^ BCA Assay Kit was used to determine the protein concentration. Proteins were separated on SDS-polyacrylamide gel electrophoresis and then transferred onto polyvinylidene difluoride membranes (Millipore). Membranes were blocked in TBST (tris(hydroxymethyl) amin omethane-NaCl-Tween 20) containing 5% nonfat dried milk, and then incubated with the primary antibodies at 4°C overnight, respectively. After that, the secondary antibodies were conjugated with horseradish peroxidase at 1:1000 dilutions for 1 h at room temperature. After that, the protein bands were stained by ECL™ Start Western Blotting Detection Reagent and visualized using ChemiDocTM XRS+ Imaging System.

### Cell cycle arrest

Cell cycle arrest was evaluated by staining DNA with PI. A549 cells were plated in 6-well plate and incubated for 12 h. Ru1 at different concentrations were then added into the wells and incubated for 24 h. After incubation, cells were collected and fixed in 1.5 ml aqueous ethanol (75%, v/v) at 4°C overnight, and then stained with PI (50 μg/mL) in the presence of RNAase A (100 μg/mL) for 30 min at 37°C in the dark. The stained cells were then analyzed by using the flow cytometer.

### Mitochondrial dysfunction

Evaluation of mitochondrial depolarization was performed by measuring changes in the MMP through three different methods, that is: flow cytometry analysis, microscopic and TEM observation. For microscopic observation, after treated with Ru1, cells were incubated in complete medium containing 10 μg/mL JC-1 for 30 min and washed twice with PBS, and then analyzed using an inverted fluorescence microscope. For flow cytometry analysis, after incubation, the cells were trypsinized and washed twice with PBS, and then incubated in 500 mL PBS containing 10 μg/mL JC-1 at 37°C in the dark for 30 min. The cells were resuspended in PBS and then analyzed by a flow cytometer. The percentage of the green fluorescence from JC-1 monomers was used to show the cells that lost MMP. For TEM observation, after incubation, cells were harvested and fixed in a 4% glutaraldehyde for 12 h at 4°C. After washed three times with PBS, the fixed cells were then incubated with 1% osmium tetroxide. Cells were then gradient dehydrated with different concentrations of ethanol and then embedded in Spurr's resin for 1 h, and incubated at 80°C for 48 h. The ultrathin sections (60 nm) cut by an ultramicrotome (Leica UC7), were post-stained with 4% uranyl acetate for 10 min and lead citrate for 1.5 min, and visualized in an electron microscope (JEM-1400, JEOL, JAPAN).

### Cellular-Ru1 distribution

Cell Mitochondria Isolation Kit was employed to detect the subcellular distribution of ruthenium. Briefly, after incubation with different concentrations of Ru1 for 24 h, cells were harvested and washed twice with PBS, and then resuspended in the mitochondria isolation buffer for 15 min. The suspension was homogenized using a Dounce homogenizer, and the homogenate was then centrifuged at 600 × g for 10 min at 2–8°C. The pellets (nuclear fraction) were completely digested in 4 mL mixture (3 mL, 95% HNO_3_ and 1 mL, H_2_O_2_), and the supernatant was centrifuged at 11 000 × g for 10 min at 2–8°C. The resulting supernatants (cytoplasmic fractions) and pellets (mitochondrial fractions) were also digested in 4 mL mixture, respectively. Finally, ruthenium element was determined by ICP-MS with a 100 ng/mL ruthenium standard solution (from Aladdin) for drawing a standard line.

### ROS detection

ROS level was detected after A549 cells had been stained with DCFH-DA. For flow cytometry analysis or microplate analysis, collected cells were trypsinized and washed three times with PBS, and then incubated for 20 min with 10 μM DCFH-DA (or with 20 nM MitoTracker) in culture medium at 37°C in the dark. Cells were washed twice and resuspended in PBS, and then analyzed by a flow cytometer or microplate analyzer. When necessary, A549 cells were pretreated with 2 μM CsA for 1 h and subsequently treated with Ru1. For microscopic observation, cells were incubated for 20 min in complete medium containing 10 μM DCFH-DA and washed twice with PBS, and then photographed using an inverted fluorescence microscope. When necessary, two antioxidants (NAC = 10 mM and Tiron = 5 mM) were added firstly in medium for 1 h before Ru1 was added.

### GFP-LC3 transfection

The GFP-LC3 expression vector was obtained from Addgene. GFP-LC3 transfection of A549 cells was performed by using Lipofectamine^®^ 2000 transfection reagent (Invitrogen). Briefly, after incubated for 20 h, cells were incubated with GFP-LC3 plasmid (2 μg/mL) which was embedded in transfection reagents for 24 h, and then exposed to Ru1 for 24 h, respectively. A549 cells were fixed in 1.5 ml aqueous ethanol (70%, v/v) at 4 °C for 30 min and washed twice with PBS. GFP-LC3 puncta signals were detected by an inverted fluorescence microscope. A minimum of 300 GFP-LC3-transfected cells were counted to quantifying the puncta signals for each sample.

### AVOs detection

After incubated with Ru1 for 24 h, cells were stained with 1 μg/mL AO for 15 min, then washed twice with PBS and examined by an inverted fluorescence microscope immediately.

### Tumor xenograft in nude mice

Tumors were established by injection (s.c.) of 1 × 10^7^ A549 cells into the 5-week old BLAB/c female athymic nude mice (Guangdong Medical Laboratory Animal Center, Foshan City, P. R. China). All procedures were performed in accordance with the protocols approved by the Guangdong Medical University Laboratory Animal Centre. Mice were randomly assigned to 1 of 3 experimental groups (*n* = 5 each) and treated with Ru1 or 0.9% NaCl when the implanted tumors reached a volume of 80–100 mm^3^. Tumor volume was monitored and calculated as Length (Width)^2^/2. Ru1 was administrated i.p. to mice 1 times/3 day for 28 days.

### Immunohistochemistry

Immunohistochemistry was conducted by using the antigen retrieval protocol followed by primary antibody incubation. Tissues fixed in 4% paraformaldehyde were embedded in paraffin and were sectioned at a thickness of 4 mm. These sections were deparaffinized, hydrated and immersed three times in PBS. Then antigen was retrieved by pre-treated in the microwave for 10 min with 10 mM citrate buffer (pH 6.0). The sections were incubated in 3% H_2_O_2_ to eliminate the activity of endogenous peroxidase for 10 min at room temperature. After washed in PBS, the sections were blocked in 5% normal goat serum for 15 min at room temperature. Slides were drained and further incubated with the primary antibody anti-rat LC3-II (1:800; CST, #3868), anti-rat cleaved caspase-3 (1:200; CST, #9661), anti-rat CD-31 (1:200; Boster, BA1346) and Ki-67 (1:200; Boster, PB0065) at 4°C overnight. The sections were incubated in biotin-labeled goat anti-rabbit secondary antibody (ZSGB-BIO, SP-9001) at 37°C for 15 min, and then in horseradish peroxidase-conjugated streptavidin at 37°C for 15 min, the peroxidase was visualized with a standard diaminobenzidine/hydrogen peroxide reaction for 2 min. The sections were counterstained with hematoxylin and finally observed under a light microscope (Nikon, Japan) and photographed.

### Statistical analysis

All of the data were expressed as the mean ± SD and all assays were performed at least three times. Differences between two groups were analyzed by Student's *t*-test. Differences with *P* < 0.05 were considered statistically significant.

## SUPPLEMENTARY MATERIALS


