# Supplementary material for: The induction of autophagy against mitochondria-mediated apoptosis in lung cancer cells by a ruthenium (II) imidazole complex
Source: Oncotarget. 2016 Nov 2;7(49):80716–34. doi: 10.18632/oncotarget.13032 (PMC5348350; doi:10.18632/oncotarget.13032)
Supplement: Supplementary file 1 [file oncotarget-07-80716-s001.pdf]

The induction of autophagy against mitochondria-mediated apoptosis in lung cancer cells by a ruthenium (II) imidazole complex

Supplementary Materials

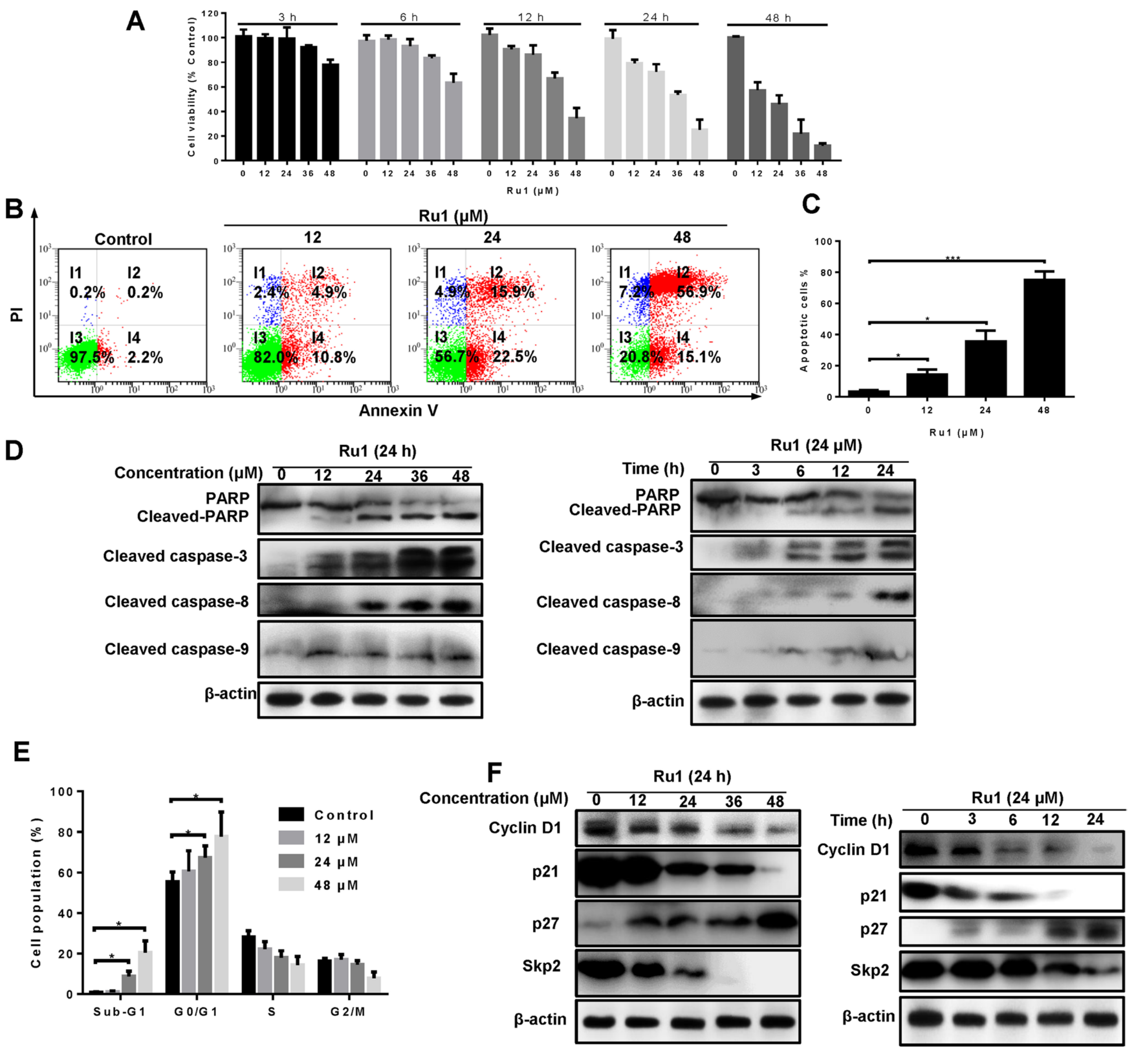

Supplementary Figure S1: Ru1 induces growth inhibition and apoptosis in NCI-H460 cells. (**A**) The NCI-H460 cell viability was measured by MTT assay after Ru1 treatment for 3, 6, 12, 24, and 48 h. (**B** and **C**) NCI-H460 cells apoptosis was detected by annexin V/PI assay after co-incubation with 12, 24, and 48 μM of Ru1 for 24 h. (**D**) The expression level of cleaved caspase-3, cleaved-PARP, cleaved caspase-8 and cleaved caspase-9 were manifested in a time- and dose-dependent manner with Ru1 treatment. (**E**) Cell cycle distribution was performed after co-incubation with Ru1 for 24 h. (**F**) The expression levels of Cyclin D1, p21, p27, and Skp2 were evaluated in a time- and dose-dependent manner with Ru1 treatment. Results were represented as mean ± SD (*$p < 0.05$, ***$p < 0.001$).

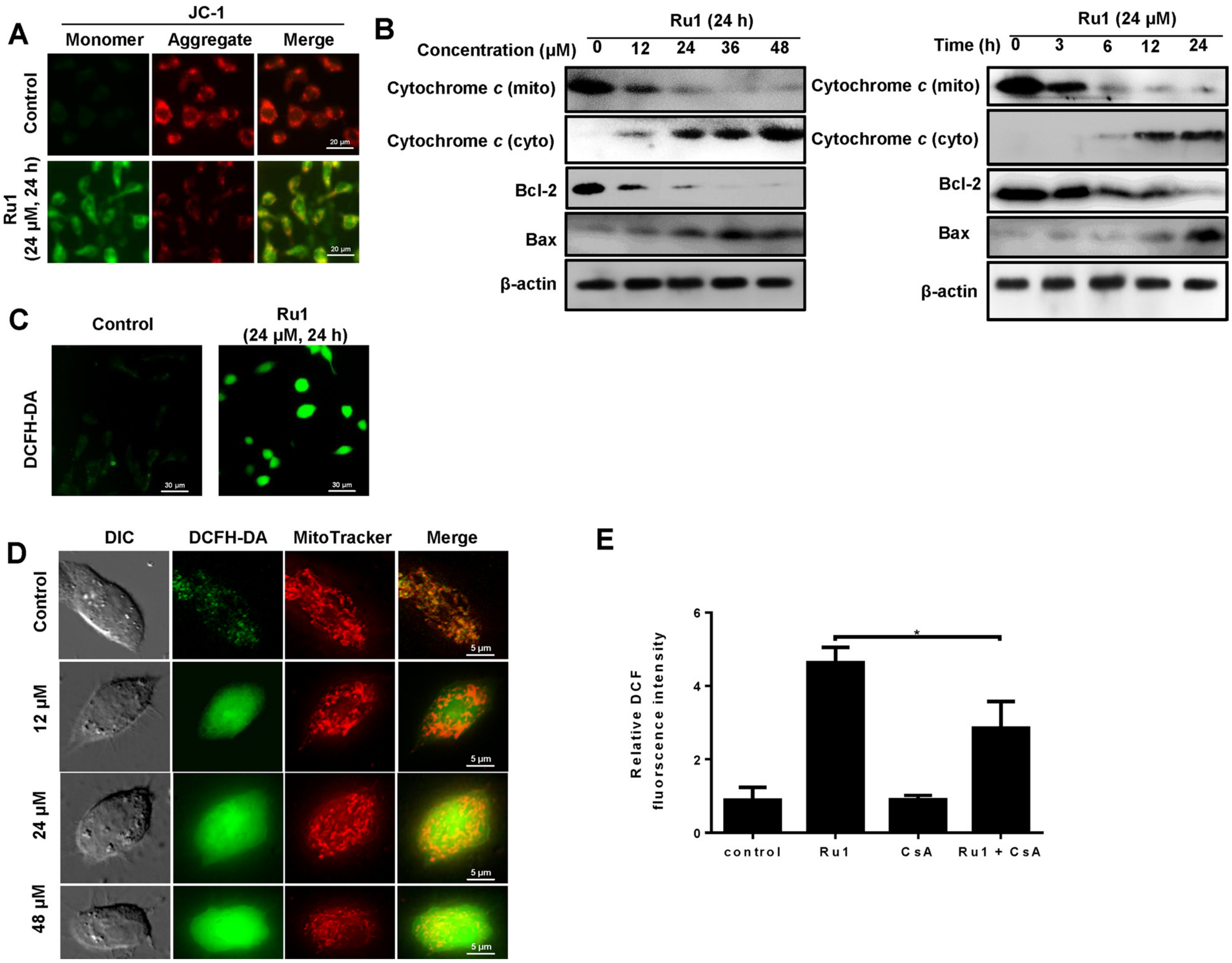

Supplementary Figure S2: Ru1 induces mitochondrial dysfunction and ROS generation in NCI-H460 cells. (**A**) Fluorescence microscope analysis of cellular MMP level by JC-1 staining after Ru1 treatment for 24 h. (**B**) The expression levels of cytochrome c, Bcl-2 and Bax were performed in a dose- and time-dependent manner. (**C**) Fluorescence microscope analysis of cellular ROS level by DCFH-DA staining after Ru1 treatment. (**D**) Ru1-triggered ROS co-localized with MitoTracker Red-stained mitochondria. Treated cells were stained with 20 nM of Mito Tracker-Red and 10 μM of DCFH-DA for 30 min. (**E**) Microplate analysis of cellular ROS level by DCFH-DA staining after 24 μM of Ru1 treatment for 24 h with, or without, 1-h CsA (2 μM) pre-treatment. Results were represented as mean ± SD (*$p < 0.05$).

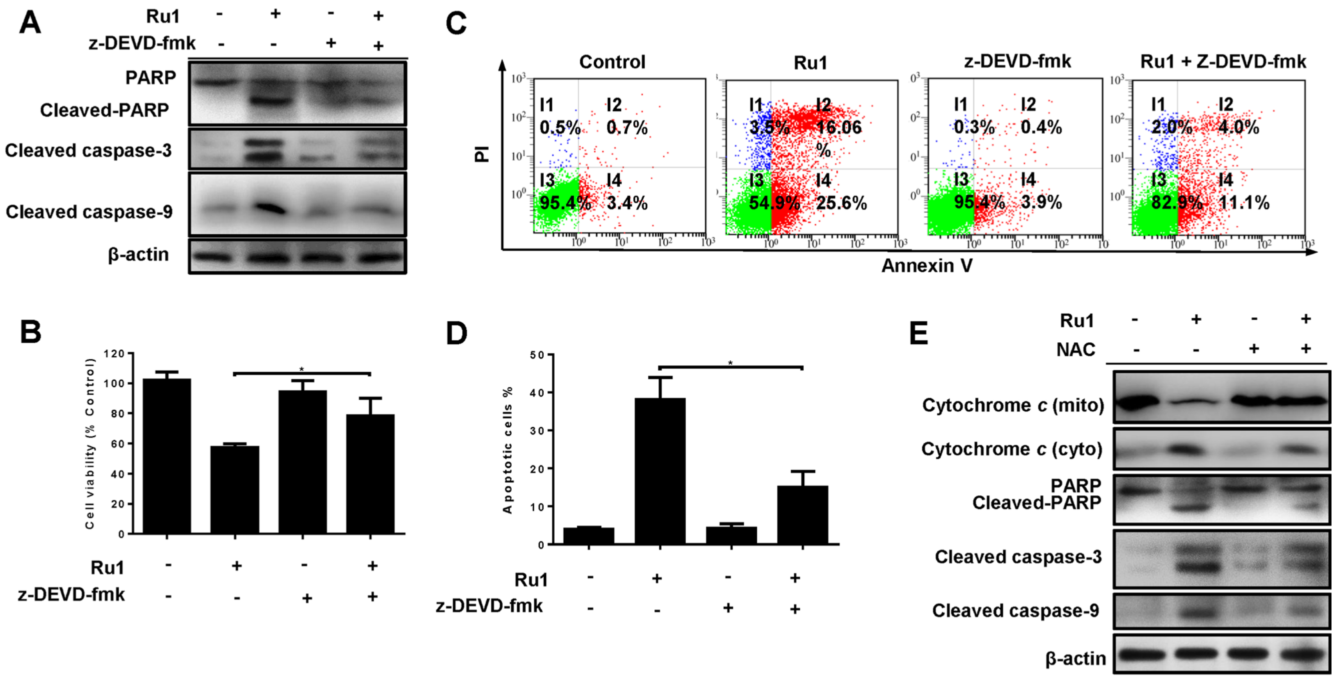

Supplementary Figure S3: Ru1-induced apoptosis is partially caspase 3-dependent by triggering ROS-mediated mitochondrial dysfunction in NCI-H460 cells. (**A**–**D**) NCI-H460 cells were incubated with Ru1 for 24 h with, or without, 1-h z-DEVD-fmk (50 µM) pretreatment. (A) The expression levels of cleaved caspase-3, PARP, cleaved-PARP and cleaved caspase-9 were assessed by western blot analysis. (B) Cell viability was measured by MTT assay. (C and D) Annexin V/PI assay was performed by flow cytometry. (**E**) The expression levels of cytochrome c, cleaved caspase-3, PARP, cleaved-PARP and cleaved caspase-9 were assessed by western blot analysis with, or without, antioxidants (NAC = 10 mM).

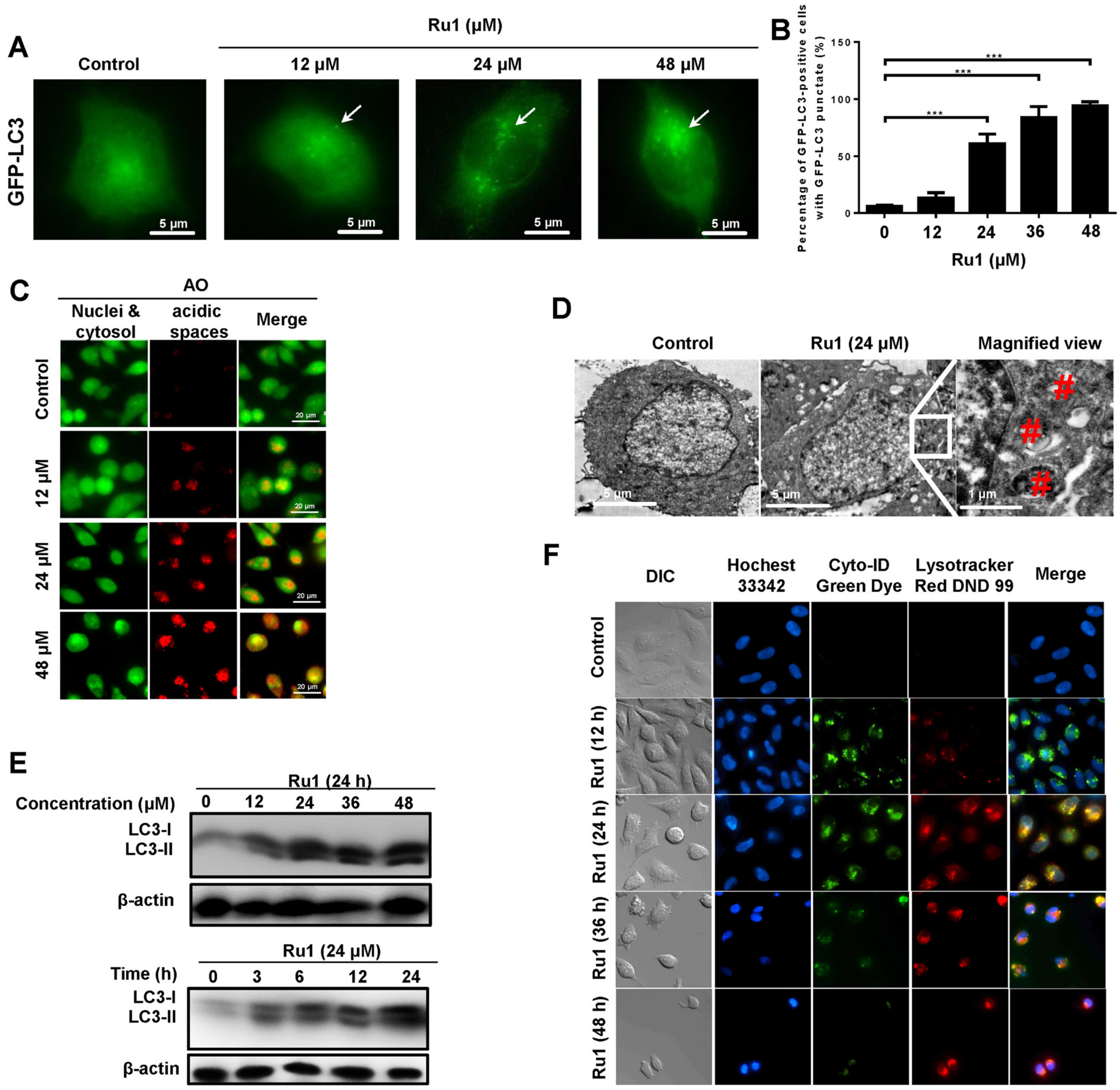

Supplementary Figure S4: Ru1 induces autophagy and autophagic flux in NCI-H460 cells. (**A**) Fluorescence microscope analysis of autophagy in NCI-H460 cells stained with GFP-LC3 after Ru1 treatment for 24 h. (**B**) GFP-LC3-postive cells were quantified in a dose-dependent manner during Ru1 treatment. Data are presented as percentages of GFP-LC3-postive cells with GFP-LC3 punctate fluorescence. (**C**) Fluorescence microscope analysis of cellular AVOs level by AO staining after different concentrations Ru1 treatments for 24 h. (**D**) TEM images showing the ultrastructure of NCI-H460 cells treated with Ru1 for 24 h. (**E**) Western blot analysis of the level of LC3-I and LC3-II in dose- and time-dependent Ru1 treatment. (**F**) Autophagic flux induced by Ru1. Cells were co-stained with Cyto-ID green dye and lysotracker red DND 99. Results were represented as mean ± SD (***$p < 0.001$).

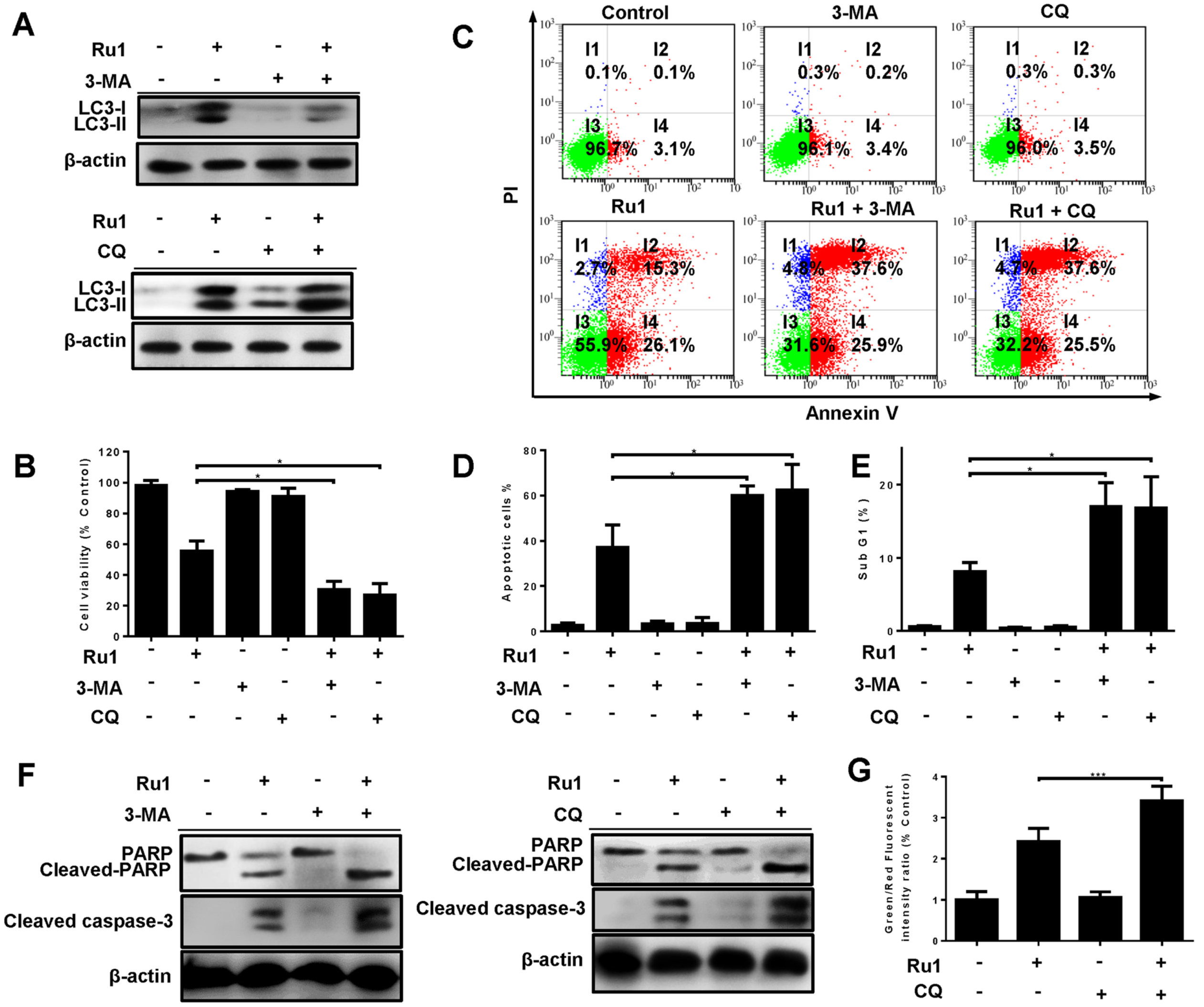

Supplementary Figure S5: Inhibition of autophagy enhances Ru1-induced growth inhibition and apoptosis of NCI-H460 cells. (**A**–**G**) Cells were pre-incubated with 10 mM 3-MA or 2.5 μM CQ for 1 h, and then exposed to Ru1 for 24 h. (A) Autophagy-associated protein LC3-I/II was detected by western blot analysis. The percentage of cell viability (B), apoptosis cells (C and D), and sub-G1 (E) were obtained by MTT assay and flow cytometry. (F and G) The expression levels of cleaved caspase-3, PARP and cleaved-PARP were assessed by western blot analysis. Results were represented as mean ± SD (*$p < 0.05$).

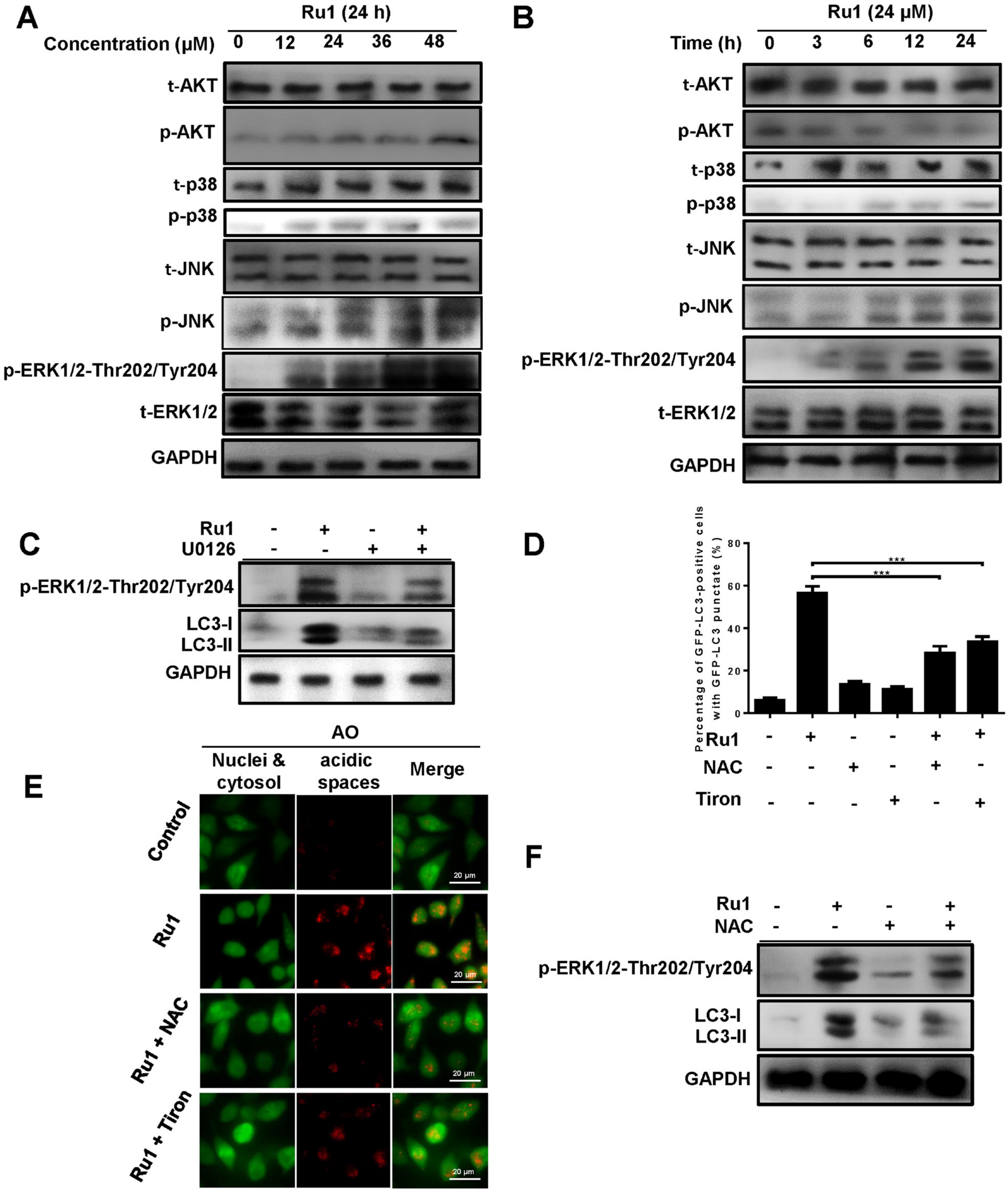

Supplementary Figure S6: The ERK signaling pathway is involved in ROS-dependent autophagy induced by Ru1 in NCI-H460 cells. (**A** and **B**) Western blot analysis of the expression level of total and the phosphorylation status MAP kinases, AKT and ERK1/2. (**C**) Western blot analysis of the levels of LC3-I/II and p-ERK1/2. NCI-H460 cells were incubated with 24 μM of Ru1 for 24 h with, or without, the ERK phosphorylation inhibitor U0126 (20 μM) pre-treatment. (**D**) GFP-LC3-postive cells were quantified after 24 μM of Ru1 treatment for 24 h with, or without, 1-h NAC (10 mM) or Tiron (5 mM) pre-treatment. (**E**) Fluorescence microscope analysis of cellular AVOs level by AO staining after 24 μM of Ru1 treatment for 24 h with, or without, 1-h NAC (10 mM) or Tiron (5 mM) pre-treatment. (**F**) Western blot analysis of the levels of LC3-I/II and p-ERK1/2. NCI-H460 cells were incubated with 24 μM of Ru1 for 24 h with, or without, 1-h NAC (10 mM) pre-treatment. Results were represented as mean ± SD (****p* < 0.001).
